# The effect of the cyclic GMP-AMP synthase-stimulator of interferon genes signaling pathway on organ inflammatory injury and fibrosis

**DOI:** 10.3389/fphar.2022.1033982

**Published:** 2022-12-05

**Authors:** Yuliang Liu, Yihui Li, Li Xue, Jie Xiao, Pengyong Li, Wanlin Xue, Chen Li, Haipeng Guo, Yuguo Chen

**Affiliations:** ^1^ Department of Critical Care Medicine, Qilu Hospital, Cheeloo College of Medicine, Shandong University, Jinan, Shandong, China; ^2^ The Key Laboratory of Emergency and Critical Care Medicine of Shandong Province, Qilu Hospital, Cheeloo College of Medicine, Shandong University, Jinan, Shandong, China; ^3^ The Key Laboratory of Cardiovascular Remodeling and Function Research, Chinese Ministry of Education and Chinese Ministry of Health, Qilu Hospital, Cheeloo College of Medicine, Shandong University, Jinan, Shandong, China; ^4^ Department of Emergency Medicine and Chest Pain Center, Qilu Hospital, Cheeloo College of Medicine, Shandong University, Jinan, Shandong, China

**Keywords:** cyclic GMP-AMP synthase-stimulator of interferon genes signaling pathway, organ fibrosis, innate immunity, remodeling, inflammation

## Abstract

The cyclic GMP-AMP synthase-stimulator of interferon genes signal transduction pathway is critical in innate immunity, infection, and inflammation. In response to pathogenic microbial infections and other conditions, cyclic GMP-AMP synthase (cGAS) recognizes abnormal DNA and initiates a downstream type I interferon response. This paper reviews the pathogenic mechanisms of stimulator of interferon genes (STING) in different organs, including changes in fibrosis-related biomarkers, intending to systematically investigate the effect of the cyclic GMP-AMP synthase-stimulator of interferon genes signal transduction in inflammation and fibrosis processes. The effects of stimulator of interferon genes in related auto-inflammatory and neurodegenerative diseases are described in this article, in addition to the application of stimulator of interferon genes-related drugs in treating fibrosis.

## Introduction

Fibrosis is a normal byproduct of chronic tissue injury and is required to regenerate of injured tissues and organs. Although fibrosis is beneficial in the short term, the long-term progression of fibrosis may cause dysfunction or even failure of cells and organs (Rockey et al., 2015; Henderson et al., 2020). Excessive fibrosis leads to abnormal remodeling and progressive dysfunction of several organs, including the heart, kidneys, lungs, liver, and other organs (Zhang and Zhang, 2020). Stress reactions and injuries (autoimmunity, sepsis, viruses, coronavirus disease 2019, inflammation, ischemia, metabolic abnormalities, toxins, *etc.*) can lead to organ fibrosis (Mack, 2018; Zhou et al., 2018; Horowitz and Thannickal, 2019; Sun Z. et al., 2020; McDonald, 2021; Ung et al., 2021). In addition, the pathophysiological process of fibrosis involves numerous signal transduction pathways, such as Ca2+, Janus kinase (JAK)/signal transducer and activator of transcription (STAT), phosphatidylinositol-3-kinase (PI3K)/protein kinase B (Akt), renin-angiotensin-aldosterone (RAAS), transforming growth factor beta (TGF-β)/Smad, wingless/integrated (Wnt)/β-catenin and many others (Mezzano et al., 2001; Goh et al., 2015; Hu et al., 2018; Feng et al., 2019; Hu H. H. et al., 2020; Montero et al., 2021; Qin et al., 2021). These signaling pathways interact to form a complex network of pathogenic mechanisms for fibrosis.

Ishikawa and Barber’s team discovered STING in 2008 and demonstrated that STING could facilitate the expression of type I interferons (IFN-1) as a defense against viral infection (Ishikawa and Barber, 2008). Since then, STING, one of the key participants in innate immunity, has gradually entered our view. Its primary physiological role is to trigger responses such as innate immunity and inflammation after recognising abnormal DNA (both exogenous pathogenic and endogenous DNA) by the cGAS (Hopfner and Hornung, 2020). Recent studies have shown that STING signal transduction occurs in the remodeling and fibrosis of various organs. The above process involves the interaction of multiple mechanisms, such as TGF-β/Smad and JAK/STAT signal transduction pathways.

There are still no effective treatments for fibrosis. Therefore, more research is essential to understand how fibrosis works and to discover viable therapeutic strategies. This article mainly explores the effects of STING and related molecules in exacerbating inflammation and promoting fibrosis in different organ diseases, exploring possible new directions for therapeutic targets. Molecular mechanisms of STING and its interactions with other signaling pathways are also included, which are extremely valuable for further clarification of the pathological process of organ fibrosis. In addition, studies of STING-related drugs were reviewed to explore their mechanisms of action and research advances. Finally, we elucidate current issues and perspectives for further research in the future.

## Summary of the cyclic GMP-AMP synthase-stimulator of interferon genes signal transduction process

### The discovery of the cyclic GMP-AMP synthase-stimulator of interferon genes signal transduction pathway

STING (or called ERIS, MITA, MPYS, TMEM173) (Jin et al., 2008; Zhong et al., 2008; Ishikawa et al., 2009; Sun et al., 2009), which is located in the endoplasmic reticulum (ER), was first reported by Ishikawa and Barber’s team in 2008. It contributes to activating interferon regulatory factor 3 (IRF3) and nuclear factor-kappa B (NF-κB) to increase IFN-1 production and thus resist virus attack (Ishikawa and Barber, 2008). Zhong et al. further demonstrated the vital effect of TANK binding kinase 1 (TBK1) in STING-induced IRF3 activation (Zhong et al., 2008). In 2013, Wu and Sun et al. discovered that cyclic guanosine monophosphate-adenosine monophosphate (cyclic GMP-AMP, or cGAMP) in mammalian cells could act as an endogenous second messenger to sense cytoplasmic DNA, thereby triggering STING and downstream interferon production (Wu et al., 2013). In the same year, they identified a new DNA sensor in the cytoplasm called cGAS, which identifies cytoplasmic DNA and promotes cGAMP production, thereby activating STING (Sun et al., 2013). At this point, the overall structure of the cGAS-STING signal transduction path was understood, and new studies related to its more specific perspectives have begun to emerge one after another.

### Activation and physiological effects of the cyclic GMP-AMP synthase-stimulator of interferon genes signal transduction pathway

As mentioned above, the initiation of the STING signal transduction needs to be triggered by cytoplasmic DNA. Sources of cytoplasmic DNA include bacteria, viruses, tumor cells, micronuclei, damaged mitochondria, *etc.* cGAS senses DNA from the above sources and undergoes conformational changes, thereby catalyzing the synthesis of the second messenger cGAMP containing 2′-5′ and 3′-5′ phosphodiester bonds from ATP and GTP (Ablasser et al., 2013; Gao et al., 2013; Zhang et al., 2013). Notably, the way cGAS recognizes DNA is highly correlated with DNA length, which means that only DNA of a certain length can effectively activate cGAS and promote the production of IFN (Andreeva et al., 2017; Luecke et al., 2017). It was shown that STING located in the endoplasmic reticulum had bound a large amount of TBK1 before being activated, and these TBK1 were also in an equally inactive state (Zhang et al., 2019). The conformation of STING changed after binding to cGAMP. The structural site used to bind the ligand rotates 180° clockwise relative to the transmembrane part and releases the C-terminal tail. Eventually, multiple STING molecules in parallel undergo oligomerization and form disulfide bonds on cysteine residue 148 to stabilize the structure (Ergun et al., 2019; Shang et al., 2019). At the same time, STING, which binds cGAMP, exits the ER and translocates to the ER-Golgi intermediate compartment (ERGIC) and the Golgi. This is a coat protein complex II (COP II)-dependent process and is also regulated by ADP-ribosylation factor (ARF) GTPases (Dobbs et al., 2015; Gui et al., 2019). TBK1 bound to STING is approached by forming a STING polymer and eventually activated by trans-autophosphorylation (Zhang et al., 2019; Zhao et al., 2019). Activated TBK1 catalyzes the phosphorylation of serine residues in the pLxIS motif on the C-terminal tail domain of STING, thereby allowing IRF3 to be recruited and bound to this motif (Liu et al., 2015). At this time, the neighboring TBK1 phosphorylates and dimerizes IRF3, which then regulates gene expression to generate IFN-1. Furthermore, inhibitor of kappa B kinase (IKK) is also recruited by STING to activate downstream NF-κB and facilitate the synthesis of pro-inflammatory cytokines such as interleukin-1 (IL-1), interleukin-6 (IL-6) and tumor necrosis factor-alpha (TNF-α). Notably, it remains undetermined whether this process requires the mediating role of TBK1 (Konno et al., 2013; Abe and Barber, 2014; Fang et al., 2017; de Oliveira Mann et al., 2019; Balka et al., 2020).

Based on previous research, the effective range of cGAS-STING has been extended to include all aspects of resistance to infection by pathogenic microorganisms (bacteria, viruses, parasites), anti-tumor, fibrosis, immunity, and inflammation. Inflammation is closely associated with fibrosis, and the former is often the initiating factor of the latter (Mack, 2018). Pro-inflammatory cytokines can be generated by the activated STING pathway and promote the onset and spread of inflammation. It can, for example, promote the conversion of endothelial cells, which are one of the vital participants in the formation and spread of inflammation (Zhao et al., 2021), into a pro-inflammatory phenotype (Pober and Sessa, 2007). In addition, activated endothelial cells can produce abundant chemokines and recruit mononuclear macrophages (Butcher, 1991) capable of producing inflammation-associated cytokines and TGF-β (Arabpour et al., 2021). Previous extensive conclusions have shown that TGF-β is conducive to the multiplication of fibroblasts and the aggregation of ECM, such as collagen (Fine and Goldstein, 1987; Clark et al., 1997). The above is only one aspect of STING-driven inflammation and fibrosis, and more specific molecular mechanisms remain to be added.

### cyclic GMP-AMP synthase-stimulator of interferon genes is one of the interconnected and interacting pro-fibrotic signal pathways

TGF-β signal transmission contributes to fibrogenesis (Meng et al., 2016). TGF-beta receptor II (TGF-β RII) is activated by autophosphorylation after binding to TGF-β. TGF-β RII then recruits and phosphorylates TGF-beta receptor I (TGF-β RI), the next factor that activates Smad2/3. During this process, Smad4 forms a trimer with activated Smad2/3. It is transported to the nucleus, thereby enhancing the synthesis of a range of molecules associated with fibrosis, including fibronectin, alpha-smooth muscle actin (α-SMA), collagen, and so on (Heldin and Moustakas, 2016; Meng et al., 2016; Hu et al., 2018). In addition, the above processes facilitate the transformation of non-myofibroblasts into myofibroblasts and the activation of myofibroblasts, thus promoting the deposition of ECM (Mack and Yanagita, 2015; Meng et al., 2016). Activation of ER stress can be observed in mice receiving aortic banding or angiotensin II-induced cardiomyocytes, and in mice receiving CCl_4_ (Iracheta-Vellve et al., 2016; Zhang et al., 2020). ER stress was shown to facilitate the stimulation of the cGAS-STING axis (Zhang et al., 2020), thereby promoting the generation of IFN-1 and NF-κB. Pro-inflammatory cytokines produced by NF-κB can promote the transcription and expression of TGF-β isoforms (Villiger et al., 1993). Notably, macrophages at the site of inflammation can continue to secrete inflammation-associated cytokines or TGF-β (Arabpour et al., 2021). In addition, angiotensin II can also promote the generation of TGF-β (Liu et al., 2017). As a result of the above interactions, the cGAS-STING signaling axis establishes an interconnection with the TGF-β/Smad signal transduction process, which is involved in fibrogenesis.

Numerous studies have confirmed that the JAK/STAT signal transduction is also closely relevant to the fibrotic process in many diseases, such as diabetic nephropathy (Zhang et al., 2021). The cGAS-STING signaling pathway produces interleukins and interferons that can function as ligands for the JAK/STAT signaling pathway. The receptor undergoes dimerization upon attachment to the ligand. JAK then couples to the receptor and undergoes phosphorylation activation, which then recruits and phosphorylates STAT, at which point the phosphorylated STAT forms a dimer and enters the nucleus, thereby regulating transcription and expression of the relevant genes (Morris et al., 2018; Bharadwaj et al., 2020; Montero et al., 2021; Zhang et al., 2021).

**FIGURE 1:**
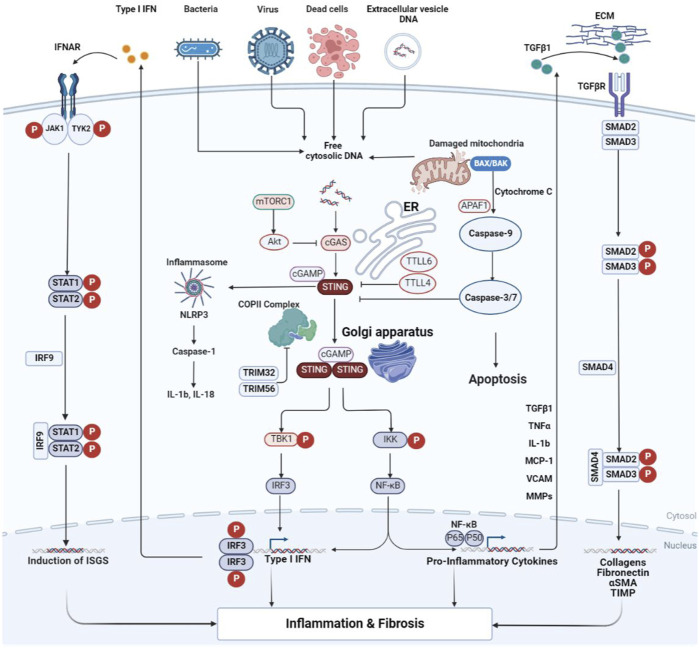
Crosstalk of the cGAS-STING signal transduction during inflammation and fibrosis. Cytoplasmic DNA from bacteria, viruses, dead cells, mitochondria, and extracellular vesicles is recognized by cGAS and thus activates STING. STING then exits the ER and transfers to the Golgi apparatus. During this process, STING completes the recruitment of TBK1 and IKK, as well as the activation of IRF3 and NF-κB. TGF-β activates TGF-β receptors, resulting in phosphorylation of Smad2/3. The activated Smad2/3 enters the nucleus to promote transcription of fibrosis-related genes. The pro-inflammatory cytokines produced by NF-κB can act on the signal transmission process of TGF-β/Smad. IFN from the STING pathway activates the IFN receptor, which is sequentially phosphorylated by the receptor-coupled JAK as well as downstream STAT, and the phosphorylated STAT dimer transfers to the nucleus to modulate gene transcription. cGAS is subject to negative regulatory effects induced by mTORC1 and Akt. It can also be inhibited in activity by TTLL4 and TTLL6, thus antagonizing the activation of STING. The apoptosis of the Bax pathway is initiated by caspase-9 and executed by caspase3/7, thus inhibiting the effect of cGAS initiation. α-SMA, alpha smooth muscle actin; Akt, protein kinase B; APAF1, apoptotic protease activating factor-1; BAX, BCL2-associated X protein; cGAMP, cyclic GMP-AMP; cGAS, cyclic GMP-AMP synthase; COP II, coat protein complex II; ECM, extracellular matrix; ER, endoplasmic reticulum; IFN, interferon; IFNAR, the interferon-α/β receptor; IKK, inhibitor of kappa B kinase; IL, interleukin; IRF, interferon regulatory factor; ISGs, interferon-stimulated genes; JAK1, janus kinase 1; MCP-1, monocyte chemoattractant protein-1; MMPs, matrix metalloproteinases; mTORC1, mechanistic target of rapamycin complex 1; NF-κB, nuclear factor-kappa B; NLRP3, NOD-like receptor thermal protein domain associated protein 3; STAT, signal transducer and activator of transcription; STING, stimulator of interferon genes; TBK1, TANK binding kinase 1; TGF-β, transforming growth factor beta; TGFβR, transforming growth factor beta receptor; TIMP, tissue inhibitor of metalloproteinase; TNF, tumor necrosis factor; TRIM, tripartite motif; TTLL, tubulin tyrosine ligase-like enzymes; TYK2, tyrosine kinase 2; VCAM, vascular cell adhesion molecule.

As a possible inducing factor of fibrosis, apoptosis is closely related to STING (Iracheta-Vellve et al., 2016). BCL-2-associated X protein (BAX) is an apoptosis-promoting protein involved in the mitochondrial apoptotic pathway, and its levels can be upregulated by STING (Li A. et al., 2019). On the one hand, BAX increases the permeability of the mitochondrial membrane to induce the leakage of cytochrome C and then initiates apoptosis through caspase-9 and downstream caspase-3/7 (Murthy et al., 2020; Xiong et al., 2021). On the other hand, mitochondrial DNA (mtDNA) released from mitochondria can activate cGAS. In addition, ER stress is also involved in promoting the interconnection between STING and BAX (Murthy et al., 2020). STING can also activate the MAPK pathway, but the exact mechanism remains to be investigated (Hopfner and Hornung, 2020). From the information available to date, the pro-fibrotic effects of STING are achieved, at least in part, through several of the classical fibrotic pathways described above. Activated STING mediates inflammatory tissue damage, thereby inducing the process of tissue repair and fibrosis. The TGF-β pathway is an essential player in the latter, actively responding to the STING-induced inflammatory microenvironment. In addition, STING activates numerous downstream effector molecules (e.g., NF-κB, IL-6, NLRP3, *etc.*) that synergistically promote inflammatory responses, thereby exacerbating tissue damage (Li N. et al., 2019). To summarize, cGAS-STING forms an intricate network with numerous molecules and mechanisms involved in the fibrosis process (the above process is summarized in Figure 1).

## The cyclic GMP-AMP synthase-stimulator of interferon genes signal path is a mediator of inflammatory and fibrotic processes in numerous diseases

### The aberrantly activated cyclic GMP-AMP synthase-stimulator of interferon genes axis drives autoimmune and autoinflammatory diseases

STING-mediated physiological effects are critical for fighting pathogenic microorganisms such as viruses and bacteria and maintaining normal host immune function. However, excessive stimulation of cGAS and STING for various reasons can also lead to autoimmune and autoinflammatory diseases.

Aicardi-Goutières syndrome (AGS) can be caused by mutations in the human three-prime repair exonuclease 1 (*Trex1*) gene, which is essential for DNA degradation and preventing cytoplasmic DNA accumulation (Gall et al., 2012; Gao et al., 2015). Previous findings have suggested that intact *Trex1* negatively regulates STING-mediated effects. Fibromyositis of the heart occurs in *Trex1*-deficient mice, especially extensive fibrosis near the endocardium. In addition, fibromyositis also occurs in the skeletal muscles and tongue (Gall et al., 2012). In addition, mutations in *Trex1* have previously been shown to be highly relevant to familial chilblain lupus (FCL), retinal vasculopathy with cerebral leukodystrophy (RVCL), and systemic lupus erythematosus (SLE) (Lee-Kirsch et al., 2007; Rice et al., 2007; Rice et al., 2015). In recent years, elevated levels of cytoplasmic double-stranded DNA (dsDNA), IFN-1, and ISGs have been found in SLE patients, suggesting start-up of the cGAS-STING axis (Gkirtzimanaki et al., 2018; Kato et al., 2018; Wang et al., 2018). IFN-1 is an important link in the progression of SLE and is secreted mainly by dendritic cells and plasma cells (Murayama et al., 2020; Thim-Uam et al., 2020). Similarly, a dNTPase named sterile alpha motif and HD domain-containing protein 1 (SAMHD1) activates the exonuclease activity of MRE11 and thus participates in DNA replication, contributing to the degradation of part of the nascent DNA. In contrast, mutations in SAMHD1 cause chronic stimulation of STING, which is critical for AGS pathogenesis (Coquel et al., 2018). Mutations or deficiencies of the ribonuclease H2 (*RNASEH2*) gene will affect the integrity of DNA, which in turn leads to AGS (Mackenzie et al., 2016; Aditi et al., 2021). In addition, there are still many mutations in genes that are associated with AGS, all of which play a role in nucleic acid metabolism and are not described here.

Overactivation of the STING signal transduction and downstream overproduction of interferon are thought to contribute to autoinflammatory diseases. For example, functionally acquired mutations in STING are directly related to STING-associated vasculopathy with onset in infancy (SAVI). Its main manifestations include rashes, vasculitis, interstitial lung disease, and so on (Liu et al., 2014). These manifestations are similar to the clinical features of COPA syndrome (Volpi et al., 2018). COPA syndrome is an autoimmune disease caused by COPI coat complex subunit alpha (*COPA*) mutations with persistent release and accumulation of IFN-1 (Lepelley et al., 2020). COPA is indispensable for vesicular transport from the Golgi to the ER (Brandizzi and Barlowe, 2013), so mutations in *COPA* cause STING to stay in the Golgi and overstimulate IFN-1 production (Deng et al., 2020; Lepelley et al., 2020). The above results are sufficient to demonstrate that overstimulation of STING deviates from normal immune function and thus leads to adverse effects.

### The role of stimulator of interferon genes in cardiac remodeling and fibrosis

Cardiac fibrosis is the terminal form of almost all types of heart disease (Czubryt and Hale, 2021), and STING is interspersed with fibrosis in various cardiac diseases. It has been shown that STING expression is increased in the hearts of dilated cardiomyopathy and hypertrophic cardiomyopathy patients, and in mice with myocardial remodeling caused by aortic banding. In addition, knockout of STING exhibited reduced levels of fibrosis biomarkers such as α-SMA, collagen type I (Col I), and collagen type III (Col III), and ER stress may be associated with this process (Zhang et al., 2020). Similarly, in cardiac failure mice induced by transverse aortic contraction, the above gene expression did not show an increase after cGAS knockout, revealing the effect of STING in pathological remodeling caused by cardiac pressure overload (Hu D. et al., 2020). It has also been demonstrated that the knockdown of STING reduces myocardial injury caused by STING-IRF3-activated NLRP3 (Li N. et al., 2019). The above studies revealed that the cGAS-STING path is an important participant in several heart diseases and tends to aggravate their damage, while inhibition of this signal axis may reduce the extent of fibrosis.

### Stimulator of interferon genes in pulmonary inflammation and fibrosis

Pulmonary fibrosis exhibits a high morbidity and mortality rate in various diseases. It severely impairs lung function and dramatically affects patients’ quality of life. However, few effective treatments are still available, so the search for new biomarkers and therapeutic strategies is essential. Current research suggests that the pro-fibrotic effects of STING are seen in a variety of lung diseases. Researchers used the induction of silica to create lung inflammatory injury and fibrosis in mice. They observed cleavage of caspase-3 and gasdermin D (GSDMD) and phosphorylation of mixed-lineage kinase domain-like protein (MLKL). This demonstrates that silica-treated lung cells die by apoptosis, pyroptosis, and necroptosis. The dead cells consequently leak dsDNA, which triggers the action of STING and subsequent INF-1 (Benmerzoug et al., 2018). Similarly, the lungs of mice exposed to cigarette smoke are injured and release dsDNA that initiates the process of cGAS-STING signal transduction, which aggravates inflammatory injury and fibrosis in the lungs, providing a new possible therapeutic idea for COPD (Nascimento et al., 2019). Bleomycin (BLM), an anticancer drug, is often used to induce pulmonary fibrosis. According to some research, STING is how BLM causes pulmonary fibrosis to develop. After BLM treatment, the release of dsDNA was increased and activated the STING-mediated type 1 interferon response (Seo et al., 2021). This process involves the TGF-β/Smad path, as evidenced by elevated phosphorylated Smad2/3, α-SMA and Col-1 (Shi et al., 2022). Furthermore, STING expression was positively correlated with TGF-β-induced fibrosis (Sun S. C. et al., 2020), indicating a synergistic effect of STING and TGF-β in the fibrogenesis process. The application of some materials, such as graphitized multi-walled carbon nanotubes (GMWCNTs) in medicine can also induce inflammation and fibrosis of the lungs, showing thickening of the alveolar wall and upregulation of STING, TGF-β, and collagen levels in the diseased lungs (Han et al., 2021). The development of the coronavirus disease 2019 (COVID-19) has also been mentioned to involve the STING signaling-mediated type I interferon response (Neufeldt et al., 2022). Mitochondrial dysfunction, mtDNA release, cell fusion, and nucleus disruption induced by severe acute respiratory syndrome coronavirus two infection largely contribute to the activation of this pathway (Ren et al., 2021; Zhou et al., 2021; Liu X. et al., 2022; Domizio et al., 2022). While early INF-1 helps to fight viral infection, late-stage IFN-1 has been shown to exacerbate the severe COVID-19 inflammatory response and may be associated with poor prognosis (Wang N. et al., 2020; Lee et al., 2020; Lee and Shin, 2020; Galani et al., 2021). Both tissue damage and many inflammation-associated cytokines contribute to fibrosis after COVID-19. As well as the above traditional cGAS-STING-TBK1-IRF3 axis, Zhang et al. reported a non-classical STING-protein kinase RNA-like endoplasmic reticulum kinase (PERK)-eukaryotic initiation factor 2 alpha (eIF2α) signaling pathway, which is independent of each other and the direct activation of PERK by STING precedes that of TBK1-IRF3. The activation of PERK-eIF2α can be observed in mice with BLM-induced pulmonary fibrosis, and either knockout of STING or inhibition of eIF2α can reduce pulmonary fibrosis (Zhang et al., 2022). This provides a new possibility for a therapeutic approach targeting STING.

### Stimulator of interferon genes and fibrosis in liver diseases

Fibrosis can be the common outcome of chronic liver disease, regardless of the cause (Iracheta-Vellve et al., 2016). Illness and fibrosis often characterise liver injury from various causes (such as nonalcoholic steatohepatitis and hepatitis virus) (Kisseleva and Brenner, 2021). Nonalcoholic steatohepatitis (NASH) can be considered as one of a series of types of nonalcoholic fatty liver disease (NAFLD), and its characteristics include liver inflammatory reaction, steatosis, damage to hepatocytes, and varying levels of fiber deposition (Friedman et al., 2018; Schuster et al., 2018; Pierantonelli and Svegliati-Baroni, 2019). In mice with these diseases, STING deficiency or disruption reduced hepatic steatosis, inflammatory damage and fibrous deposition (Luo et al., 2018), as indicated by the down-regulated Col1A1 and α-SMA contents (Yu et al., 2019). In addition, researchers analyzed liver samples from NASH patients and found that overall levels of STING and phosphorylated TBK1 were significantly elevated and consistent with the severity of fibrosis. At the same time, hepatic stellate cells (HSCs) were activated, and the overall levels of Col1A1, fibronectin, and TGF-β1 were significantly upregulated (Wang X. et al., 2020). Infection with schistosomiasis is also accompanied by liver fibrosis (He et al., 2020). Liang et al. used western blotting to detect fibrosis indicators in mice parasitized by Schistosoma japonicum and found that cGAS knockout mice showed less severe and a smaller proportion of fibrosis than control mice. Notably, STING knockout mice did not exhibit the above results, leading the researchers to conclude that neither STING nor IFN-β is involved in the fibrotic process in the liver following Schistosoma japonicum infection (Liang et al., 2022). Initiation of the STING signal transduction is attributed to the leakage of mtDNA to the outside of the mitochondria, followed by upregulation of pro-inflammatory cytokine expression by NF-κB, which ultimately promotes fibrosis (Yong et al., 2021; Shen et al., 2022). Additionally, liver damage and fibrosis caused by microbial DNA can also be mediated by STING signaling (Luo et al., 2022). The activation and conversion of HSCs to myofibroblasts has been shown in numerous studies to be crucial for the formation of fibrosis (Tsuchida and Friedman, 2017), revealing the role of STING activation of HSCs in fibrosis. In summary, STING may be one of the markers of liver fibrosis progression and a target for treatment.

### Stimulator of interferon genes and kidney disease

Fibrosis of the kidney is the universal pathological manifestation of chronic renal disease (Romagnani et al., 2017). It can eventually destroy the kidney’s normal function and seriously threaten patients’ health. For mitochondrial DNA to remain intact, transcription factor A (*TFAM*) is essential. Enhanced cGAS-STING signaling was observed in kidney samples deficient in *TFAM*, accompanied by poor renal function, high levels of inflammation, and fibrosis, suggesting that STING-mediated renal fibrosis can be induced by mtDNA (Allison, 2019; Chung et al., 2019). Hao et al. studied a folic acid-induced fibrosis model in mice with renal insufficiency and found that the overall levels of STING, TBK1, IRF3, and perforin were elevated in IL-2-treated NK cells. After treatment with STING inhibitors and high-dose Shenkang injection, the levels of these substances decreased significantly and were accompanied by an improvement in renal function. In addition, similar results from *in vitro* experiments verified the above findings. It is proven that Shenkang injection can exert its anti-fibrosis effect by inhibiting the STING pathway (Hao et al., 2022). The increased expression of STING, TBK1 and IRF3 could also be seen in the renal tubular epithelial cells of G2APOL1 (G2 coding variant of apolipoprotein L1) mice, while the collagen deposition decreased significantly after knockout of STING. Similarly, STING inhibitors showed protective effects on renal function, such as reducing the concentration of serum creatinine, blood urea nitrogen, proteinuria, and the degree of fibrosis (Wu et al., 2021). Furthermore, some studies have found that in rhabdomyolysis-induced acute kidney injury mice deficient in absent in melanoma 2 (*AIM2*), dsDNA released from injured muscle cells is turned to activate STING and increase the expression of IFN-1 and NF-κB, exacerbating inflammation and fibrosis due to the inability of *AIM2* deficiency to scavenge inflammatory macrophages in time (Baatarjav et al., 2022).

### The relationship between stimulator of interferon genes and neurodegenerative diseases

In recent years, some new achievements have been made in researching the cGAS-STING axis in degenerative neurologic disorders. Alzheimer’s disease (AD) is a prevalent neurologic degenerative condition, and its pathological manifestations are mainly characterized by amyloid beta-peptide (Aβ) plaques and tau neurofibrillary tangles (Jack et al., 2018; Scheltens et al., 2021). In addition, more and more studies suggest that neuroinflammation is a key driver of AD progression. Hou et al. conducted a study using an APP/PS1 mutant mouse model of AD and used the nicotinamide adenine dinucleotide (NAD) precursor nicotinamide riboside (NR) as a treatment and found significantly elevated levels of NLRP3 inflammasome in APP/PS1 mutant mice. And both DNA damage and the degree of inflammation were improved by using NR. Moreover, STING and cGAS expression was increased in microglia, and NR decreased cytoplasmic DNA levels in human AD fibroblasts, suggesting that neural inflammation may be induced by signaling associated with cGAS and STING (Hou et al., 2021).

Parkinson’s disease (PD) is another common neurologic degenerative condition. Both in the intrastriatal αSyn preformed fibril (αSyn-PFF) mouse model of PD and in microglia cultured *in vitro*, αSyn-PFFs caused DNA damage, as evidenced by increased levels of γH2A.X, a marker of DNA damage. Furthermore, activation of TBK1 in mice and increased STING expression in human Parkinson’s disease patients show that STING activation contributes to α-synuclein-induced neuroinflammation and degeneration (Hinkle et al., 2022).

In mice with amyotrophic lateral sclerosis (ALS), accumulated TAR DNA-binding protein 43 in the cytoplasm can lead to mtDNA leakage and thus activate cGAS, ultimately causing upregulation of NF-κB and IFN-1 levels. In contrast, in STING-deficient ALS mice, neuroinflammation and degeneration were ameliorated (Yu et al., 2020). In addition, the amplification of the GGGGCC sequence in the open reading frame 72 of chromosome 9p (*C9ORF72*) is directly related to the pathogenesis of most familial ALS (DeJesus-Hernandez et al., 2011; Renton et al., 2011), and deletion of *C9ORF72* can promote STING-mediated IFN-1 production (McCauley et al., 2020). Superoxide dismutase 1 (*SOD1*) mutations are another common cause of inherited ALS (Chen et al., 2013; Mejzini et al., 2019), resulting in misfolding of the SOD1 protein. In *SOD1* mutant ALS mice, cGAS senses mitochondrial destruction and activates STING (Tan et al., 2022). This evidence provides a new option for effective interventions in neurodegenerative diseases, that is, to suppress STING.

### Stimulator of interferon genes and other diseases

In BLM-induced systemic sclerosis mice, an increase in cytoplasmic DNA was observed, which promoted the translocation of RNA polymerase III A (POLR3A) and activated the POLR3A/STING pathway. The result is the activation of fibroblasts, increased collagen, vascular endothelial injury, and fibrosis. Both knockout of STING and the use of H-151 (an inhibitor of STING) can reduce the fibrosis and vascular lesions of systemic sclerosis (Liu C. et al., 2022). Mouse colitis and human inflammatory bowel disease (IBD) have the characteristics of chronic inflammatory injury and fibrosis, and the high expression of STING can also be observed in their intestines (Shmuel-Galia et al., 2021). Notably, in addition to exacerbating colonic inflammation caused by intestinal microorganisms, STING promotes the expression of the cytokine IL-10, which has anti-inflammatory properties, to diminish the extent of colitis. In addition, it also reduces the production of intestinal polyps and may reduce the potential for conversion of colitis to cancer. This demonstrates the function of STING in maintaining immune homeostasis (Ahn et al., 2017). On the other hand, the mutated autophagy-related protein 16-like 1 (*Atg16l1*) gene triggers impaired autophagy, resulting in reduced STING and cytoplasmic DNA clearance, which is closely associated with IBD. Treatment with IL-22 can activate the cGAS-STING pathway by endoplasmic reticulum stress or by increasing cytoplasmic DNA, and deletion or mutation of the *Atg16l1* gene has been shown to enhance the IFN-1 levels elevated by IL-22, thereby exacerbating intestinal inflammatory injury (Aden et al., 2018). The above results reveal the association of the *Atg16l1* gene, STING and IL-22 in IBD. In addition, recent results show that in mouse ovarian granulosa cells, mtDNA can activate the STING pathway through a similar mechanism, causing an increase in cytokines such as TNF-α, thereby promoting the spread of inflammation and interstitial hyperplasia (Liu K. et al., 2022). Rheumatoid arthritis can be driven by TNF, which also relies on STING-IRF3-mediated interferon production. In THP-1 cells chronically treated with TNF, mtDNA was observed to be released into the cytoplasm and bound to cGAS to initiate a downstream inflammatory response, consistent with elevated ISGs in the joints of the mouse model (Willemsen et al., 2021).

**FIGURE 2:**
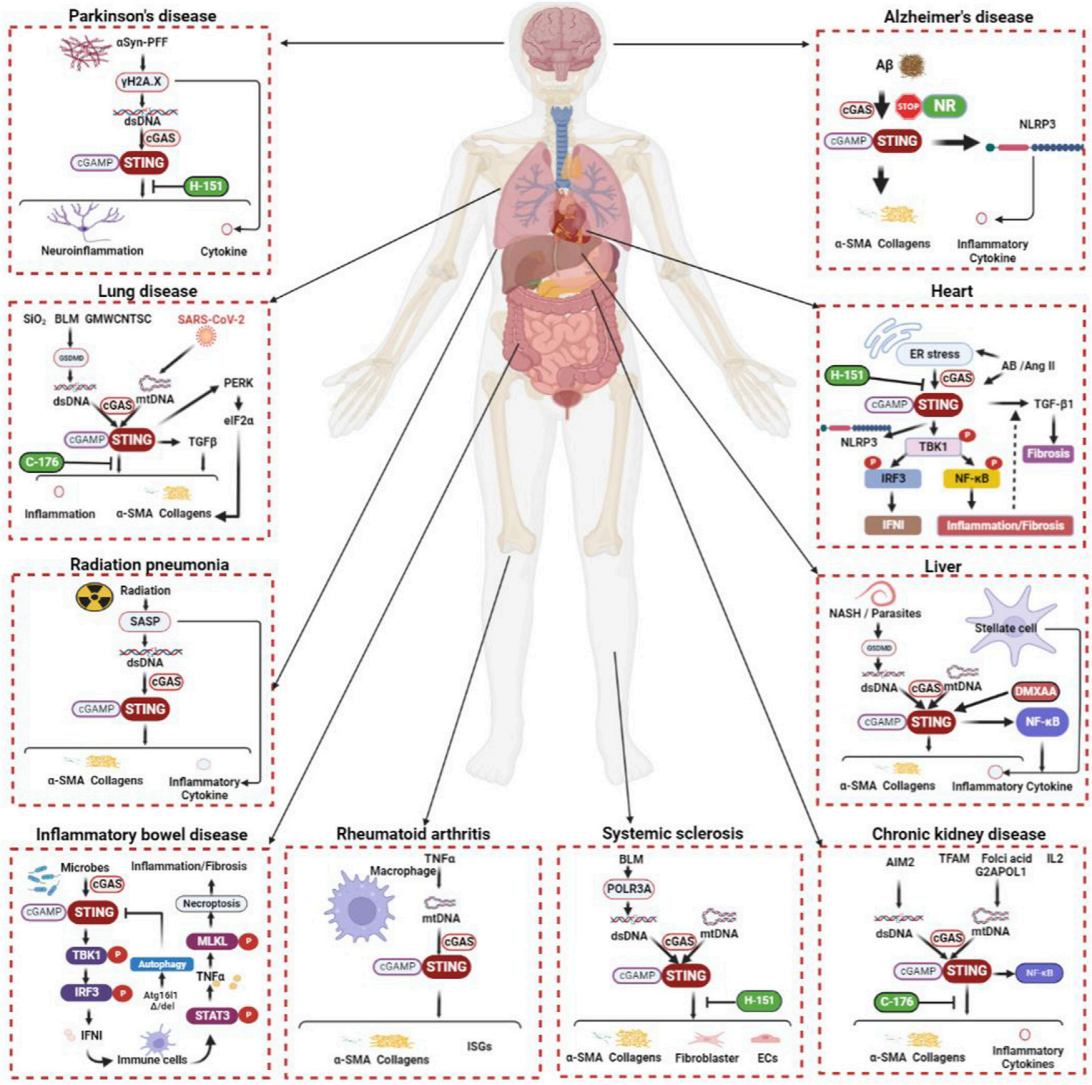
The cGAS-STING signaling pathway during organ inflammation and fibrosis. The cGAS-STING signal transduction process is inseparably linked to inflammation and fibrogenesis in multiple systems, including respiratory, circulatory, digestive, urinary, and neurological systems. The etiology of fibrosis includes inflammation, injury, toxins, radiation, foreign bodies, parasites, immunity, *etc.* STING works with TGF-β and many other molecular mechanisms to promote fibrosis in various organs. Although not all fibrosis etiologies and related mechanisms can be described exhaustively here, it is certain that the above pathways have a place in the fibrosis of different human systems. α-SMA, alpha smooth muscle actin; αSyn-PFF, αSyn preformed fibril; Aβ, amyloid beta-peptide; AB, aortic banding; AIM2, absent in melanoma 2; Ang-II, angiotensin II; Atg16l1, autophagy-related protein 16-like 1; BLM, bleomycin; cGAMP, cyclic GMP-AMP; DMXAA, 5,6-dimethylxanthenone-4-acetic acid; dsDNA, double-stranded DNA; ECs, epithelial cells; eIF2α, eukaryotic initiation factor 2 alpha; ER, endoplasmic reticulum; G2APOL1, G2 coding variant of apolipoprotein L1; GMWCNTs, graphitized multi-walled carbon nanotubes GSDMD, gasdermin D; IFN, interferon; IL, interleukin; IRF, interferon regulatory factor; ISGs, interferon-stimulated genes; MLKL, mixed-lineage kinase domain-like protein; mtDNA, mitochondrial DNA; NASH, nonalcoholic steatohepatitis; NF-κB, nuclear factor kappa-B; NLRP3, NOD-like receptor thermal protein domain associated protein 3; NR, nicotinamide riboside; PERK, protein kinase RNA-like endoplasmic reticulum kinase; POLR3A, RNA polymerase III A; SARS-CoV-2, severe acute respiratory syndrome coronavirus 2; SASP, senescence-associated secretory phenotype; STAT, signal transducer and activator of transcription; STING, stimulator of interferon genes; TBK1, TANK binding kinase 1; TFAM, mitochondrial transcription factor A; TGF-β, transforming growth factor beta; TNF, tumor necrotic factor.

Radiation therapy is an important cancer treatment, but the clinical complications it causes should not be ignored. Treating malignant tumors with radiation is often complicated by lung radiation damage, which can eventually cause pulmonary fibrosis. The activated STING signal axis exists in the macrophages of mice with radiation pneumonia (RP), suggesting a possible association with inflammation and fibrosis in RP (Yang et al., 2022a). In addition, radiation has been shown to accelerate cancer cell senescence and is associated with an elevated senescence-associated secretory phenotype (SASP). This process involves the recognition of radiation-induced dsDNA by cGAS and thus activates the subsequent effects of STING (Glück et al., 2017; Takahashi et al., 2018; Constanzo et al., 2021). Furthermore, SASP after radiation therapy is thought to promote fibrosis (Nguyen et al., 2018). This partially explains how STING contributes to fibrogenesis by radiation treatment for cancer. In Figure 2, the connection between STING and the illnesses of each of the organs mentioned above is depicted uniformly.

## Application of stimulator of interferon genes agonists and antagonists

Theoretically, STING agonists can aggravate the fibrogenic effect of cGAS-STING signaling. Therefore, STING activators are not usually used to treat fibrosis. STING agonists, such as DMXAA, have been shown to aggravate liver inflammation and steatosis in mice, as well as increase TNF-α and IL-6 expression (Yu et al., 2019). In addition, wild-type mouse BMDMs were co-cultured with human liver stellate cells in conditions containing TGF-β. When the above cells were treated with DMXAA, the phosphorylation level of p38 and the content of molecules associated with fibrous deposition (e.g. α-SMA) were increased (Luo et al., 2018). Therefore, the application of STING activators in fibrosis treatment is limited except for experimental purposes. STING agonists utilize the principle of cGAS-STING-induced type I interferon response and are now commonly used in clinical practice as one of the anti-tumor treatments (Jiang et al., 2020; Wang Y. et al., 2020; Amouzegar et al., 2021). DMXAA and ADU-S100 (an agonist of STING) attenuated motor dysfunction in mice with bone tumors and reduced abnormal behavior in mice due to bone pain. X-rays showed that bone destruction due to tumors was also ameliorated by STING agonists (Wang et al., 2021). In the BRCA1-deficient mouse breast cancer model, DMXAA also promoted the polarization of macrophages to M1 in the tumor microenvironment, thus enhancing the anti-tumor activity of macrophages (Wang et al., 2022). In a clinical trial of melanoma, breast cancer, lymphoma, ovarian cancer, and many other tumor types, ADU-S100 showed good safety and tolerance, accompanied by immune activation such as increased levels of inflammation and active T cell proliferation (Meric-Bernstam et al., 2022). In addition, the melanoma model of B16-F10 mice was treated with c-di-GMP packaged with nanoparticles, and the result was that the drug resistance of the tumor to anti-PD-1 was reduced (Nakamura et al., 2021). In addition to these, many CDN analogues or synthetic agonists still play a role in a variety of solid tumor types (Jiang et al., 2020). The types, effects, safety, mechanisms, and modes of action of these agonists will be important future research directions in tumor immunity.

As STING inhibitors, C-178 and C-176 belong to nitrofuran derivatives. The nitro and furan rings are essential for maintaining activity, and the group at the 4-position of the phenyl ring also influences the magnitude of their effects. These two compounds were shown to inhibit palmitoylation of STING by covalent modification of cysteine 91 (Cys91) (Haag et al., 2018), whereas palmitoylation of Cys88/91 is essential for STING activation (Mukai et al., 2016). In mice, C-178 and C-176 partially reversed the activating effect of CMA (a STING agonist) on STING. In addition, using C-176 reduced some clinical manifestations of AGS syndrome in *Trex1* knockout mice. However, the above two inhibitors did not show inhibitory effects on human STING (hsSTING) (Haag et al., 2018). C-176 inhibits perinuclear translocation of STING and NF-κB translocation into the nucleus, thereby reducing upregulated inflammatory cytokines and ultimately reducing fibronectin expression to reduce liver fibrosis (Shen et al., 2022). In addition, C-176 attenuated kidney fibrosis and GMWCNTs-mediated inflammatory injury and fibrous deposition in the lung, thereby protecting organ function (Chung et al., 2019; Han et al., 2021). Intestinal reperfusion injury can involve the lungs and cause acute tissue damage. C-176 can significantly reduce apoptosis in this case, thereby improving lung injury and subsequent fibrosis. Notably, STING inhibition was accompanied by an increase in AMPK phosphorylation levels, which suggests an interaction between AMPK and STING from another perspective (Yang et al., 2022b). Indole urea (H-151) is derived from 3-acylamino indole, which is also a derivative of nitrofuran. It is a selective STING inhibitor with high efficiency and a strong inhibitory effect on both mouse STING and hsSTING (Haag et al., 2018). It improves heart function in mice after myocardial infarction (MI) by reducing STING-induced type 1 interferon response and inflammation. In addition, cardiac remodeling, apoptosis of cardiomyocytes, and the generation of fibrotic biomarkers, including α-SMA, collagen, *etc.*, were reduced (Hu et al., 2022; Rech et al., 2022). However, using H-151 did not seem beneficial in improving mortality in the early stages of MI (Rech et al., 2022).

Similarly, endogenous nitro fatty acids (NO2-FA) can also reduce IFN-1 production by inhibiting the palmitoylation of STING (Hansen et al., 2018). It is a product of endogenous unsaturated fatty acids nitrated by nitrogen dioxide and has anti-inflammatory properties (Delmastro-Greenwood et al., 2015). The cyclic peptide Actin C is a chlorinated cyclopentapeptides extracted from the natural medicinal plant Aster Tataricus and belongs to one kind of Compositae cyclopeptide. It is composed of one protein amino acid and four non-protein amino acids. In addition, it also contains two chlorine atoms as functional groups. Astin C is a STING antagonist that acts at the site on STING that binds cyclic dinucleotide (CDN), and the recruitment of IRF3 by STING is thus blocked (Li et al., 2018). It is worth noting that NO2-FAs, Astin C, and tetradroisoquinolone acetic acid (compound 18, an inhibitor of STING) (Siu et al., 2019) were found to be less effective against human STING. In addition to the above, Hong et al. substituted phenoxy-methyl in a group of compounds containing benzene-1-sulfonamido-3-amide groups with phenol hydroxyl groups, resulting in the compound SN-011, a STING inhibitor with high affinity for the CDN-binding pocket, which improved systemic inflammation and clinical outcomes in *Trex1* knockout mice and was shown to be effective in both mice and humans, with lower toxicity and higher specificity than H-151 (Hong et al., 2021). ISD017 is a fusion peptide of the influenza hemagglutinin protein and is located in the N-terminal region of the HA2 peptide chain of hemagglutinin. It is another STING inhibitor effective in both mice and humans. It relies on the STING ER retention factor stromal interaction molecule 1 (STIM1) to prevent STING migration from the ER to the Golgi, thereby blocking the downstream activity (Prabakaran et al., 2021). Given that SN-011 and ISD017 are relatively new results, their detailed mechanisms and anti-fibrotic effects remain to be investigated. Details of the application of STING-related drugs can be found in the Supplementary Material.

From the currently available information, there is no doubt that STING inhibitors are effective in improving fibrosis, so more and more in-depth animal experiments and clinical trials are essential for the clinical translation of STING inhibitors.

## Discussion and prospect

According to general belief, the cGAS-STING axis assists the body in combating potentially adverse factors such as bacteria, viruses, and tumors. However, a large number of new results on the cGAS-STING signal transduction pathway that exaggerate inflammatory damage and its pro-fibrotic effects show its bad side. It is now well established that activated cGAS-STING promotes inflammatory injury and subsequent fibrotic repair, which enriches the mechanistic system of fibrosis and contributes to the search for new therapeutic strategies against fibrosis. In addition, the effects of cGAS-STING pathway activation gave us many insights. As previously mentioned, auto-inflammatory diseases characterized by STING overactivation and IFN-1 overexpression (e.g., SAVI and AGS) have more promising therapeutic targets. STING inhibitors will fundamentally block the progression of this type of disease by preventing the continued transmission of STING signals, thereby reducing excessive inflammatory damage. Inflammation and fibrosis are often two juxtaposed processes in tissue injury, which means that numerous patients with congenital autoimmune diseases would benefit from STING inhibitors, as they often suffer irreversible organ remodeling. In addition, considering the source of DNA recognized by cGAS, we believe that the administration of membrane stabilizers or drugs that protect the integrity of the nuclear membrane for specific diseases such as radiological lung injury could help to block the conduction of this pathway from upstream of STING.

However, many unknown aspects still need to be answered by future research. For instance, how to discriminate between one’s DNA and exogenous deleterious DNA, how to maintain appropriate inflammation, and how to circumvent the pathogenic effects of STING while making full use of its immune defenses are all important research directions. The signaling networks involved in fibrosis are intricate, and here we identify some of the mechanisms of STING based on existing studies. However, the specific detailed mechanisms of cGAS-STING to promote fibrosis remains to be refined, and this will be the focus of future studies. Many STING inhibitors reverse the inflammation and fibrosis of diseased organs in animal experiments. In addition, more and more STING antagonists sensitive to human STING have been screened out *in vitro*. The scope of application of STING inhibitors will expand gradually, and we believe that future clinical trials will further verify the potential of STING inhibitors.

## References

[B1] Abe T. Barber G. N. (2014). Cytosolic-DNA-mediated, STING-dependent proinflammatory gene induction necessitates canonical NF-κB activation through TBK1. J. Virol. 88 (10), 5328–5341. 10.1128/jvi.00037-14 24600004 PMC4019140

[B2] Ablasser A. Goldeck M. Cavlar T. Deimling T. Witte G. Röhl I. (2013). cGAS produces a 2'-5'-linked cyclic dinucleotide second messenger that activates STING. Nature 498 (7454), 380–384. 10.1038/nature12306 23722158 PMC4143541

[B3] Aden K. Tran F. Ito G. Sheibani-Tezerji R. Lipinski S. Kuiper J. W. (2018). ATG16L1 orchestrates interleukin-22 signaling in the intestinal epithelium via cGAS-STING. J. Exp. Med. 215 (11), 2868–2886. 10.1084/jem.20171029 30254094 PMC6219748

[B4] Aditi, Downing S. M. Schreiner P. A. Kwak Y. D. Li Y. Shaw T. I. (2021). Genome instability independent of type I interferon signaling drives neuropathology caused by impaired ribonucleotide excision repair. Neuron 109 (24), 3962–3979.e6. 10.1016/j.neuron.2021.09.040 34655526 PMC8686690

[B5] Ahn J. Son S. Oliveira S. C. Barber G. N. (2017). STING-dependent signaling underlies IL-10 controlled inflammatory colitis. Cell Rep. 21 (13), 3873–3884. 10.1016/j.celrep.2017.11.101 29281834 PMC6082386

[B6] Allison S. J. (2019). STING activation by cytoplasmic mtDNA triggers renal inflammation and fibrosis. Nat. Rev. Nephrol. 15 (11), 661. 10.1038/s41581-019-0211-y 31527791

[B7] Amouzegar A. Chelvanambi M. Filderman J. N. Storkus W. J. Luke J. J. (2021). STING agonists as cancer therapeutics. Cancers (Basel) 13 (11), 2695. 10.3390/cancers13112695 34070756 PMC8198217

[B8] Andreeva L. Hiller B. Kostrewa D. Lässig C. de Oliveira Mann C. C. Jan Drexler D. (2017). cGAS senses long and HMGB/TFAM-bound U-turn DNA by forming protein-DNA ladders. Nature 549 (7672), 394–398. 10.1038/nature23890 28902841

[B9] Arabpour M. Saghazadeh A. Rezaei N. (2021). Anti-inflammatory and M2 macrophage polarization-promoting effect of mesenchymal stem cell-derived exosomes. Int. Immunopharmacol. 97, 107823. 10.1016/j.intimp.2021.107823 34102486

[B10] Baatarjav C. Komada T. Karasawa T. Yamada N. Sampilvanjil A. Matsumura T. (2022). dsDNA-induced AIM2 pyroptosis halts aberrant inflammation during rhabdomyolysis-induced acute kidney injury. Cell Death Differ. 10.1038/s41418-022-01033-9 PMC9750976 35739254

[B11] Balka K. R. Louis C. Saunders T. L. Smith A. M. Calleja D. J. D'Silva D. B. (2020). TBK1 and IKKε act redundantly to mediate STING-induced NF-κB responses in myeloid cells. Cell Rep. 31 (1), 107492. 10.1016/j.celrep.2020.03.056 32268090

[B12] Benmerzoug S. Rose S. Bounab B. Gosset D. Duneau L. Chenuet P. (2018). STING-dependent sensing of self-DNA drives silica-induced lung inflammation. Nat. Commun. 9 (1), 5226. 10.1038/s41467-018-07425-1 30523277 PMC6283886

[B13] Bharadwaj U. Kasembeli M. M. Robinson P. Tweardy D. J. (2020). Targeting janus kinases and signal transducer and activator of transcription 3 to treat inflammation, fibrosis, and cancer: Rationale, progress, and caution. Pharmacol. Rev. 72 (2), 486–526. 10.1124/pr.119.018440 32198236 PMC7300325

[B14] Brandizzi F. Barlowe C. (2013). Organization of the ER-Golgi interface for membrane traffic control. Nat. Rev. Mol. Cell Biol. 14 (6), 382–392. 10.1038/nrm3588 23698585 PMC4064004

[B15] Butcher E. C. (1991). Leukocyte-endothelial cell recognition: three (or more) steps to specificity and diversity. Cell 67 (6), 1033–1036. 10.1016/0092-8674(91)90279-8 1760836

[B16] Chen S. Sayana P. Zhang X. Le W. (2013). Genetics of amyotrophic lateral sclerosis: an update. Mol. Neurodegener. 8, 28. 10.1186/1750-1326-8-28 23941283 PMC3766231

[B17] Chung K. W. Dhillon P. Huang S. Sheng X. Shrestha R. Qiu C. (2019). Mitochondrial damage and activation of the STING pathway lead to renal inflammation and fibrosis. Cell Metab. 30 (4), 784–799.e5. 10.1016/j.cmet.2019.08.003 31474566 PMC7054893

[B18] Clark R. A. McCoy G. A. Folkvord J. M. McPherson J. M. (1997). TGF-beta 1 stimulates cultured human fibroblasts to proliferate and produce tissue-like fibroplasia: a fibronectin matrix-dependent event. J. Cell. Physiol. 170 (1), 69–80. 10.1002/(SICI)1097-4652(199701)170:1<69::AID-JCP8>3.0.CO;2-J 9012786

[B19] Constanzo J. Faget J. Ursino C. Badie C. Pouget J. P. (2021). Radiation-induced immunity and toxicities: The versatility of the cGAS-STING pathway. Front. Immunol. 12, 680503. 10.3389/fimmu.2021.680503 34079557 PMC8165314

[B20] Coquel F. Silva M. J. Técher H. Zadorozhny K. Sharma S. Nieminuszczy J. (2018). SAMHD1 acts at stalled replication forks to prevent interferon induction. Nature 557 (7703), 57–61. 10.1038/s41586-018-0050-1 29670289

[B21] Czubryt M. P. Hale T. M. (2021). Cardiac fibrosis: Pathobiology and therapeutic targets. Cell. Signal. 85, 110066. 10.1016/j.cellsig.2021.110066 34146658 PMC8355135

[B22] de Oliveira Mann C. C. Orzalli M. H. King D. S. Kagan J. C. Lee A. S. Y. Kranzusch P. J. (2019). Modular architecture of the STING C-terminal tail allows interferon and NF-κB signaling adaptation. Cell Rep. 27 (4), 1165–1175.e5. 10.1016/j.celrep.2019.03.098 31018131 PMC7733315

[B23] DeJesus-Hernandez M. Mackenzie I. R. Boeve B. F. Boxer A. L. Baker M. Rutherford N. J. (2011). Expanded GGGGCC hexanucleotide repeat in noncoding region of C9ORF72 causes chromosome 9p-linked FTD and ALS. Neuron 72 (2), 245–256. 10.1016/j.neuron.2011.09.011 21944778 PMC3202986

[B24] Delmastro-Greenwood M. Hughan K. S. Vitturi D. A. Salvatore S. R. Grimes G. Potti G. (2015). Nitrite and nitrate-dependent generation of anti-inflammatory fatty acid nitroalkenes. Free Radic. Biol. Med. 89, 333–341. 10.1016/j.freeradbiomed.2015.07.149 26385079 PMC4684780

[B25] Deng Z. Chong Z. Law C. S. Mukai K. Ho F. O. Martinu T. (2020). A defect in COPI-mediated transport of STING causes immune dysregulation in COPA syndrome. J. Exp. Med. 217 (11), e20201045. 10.1084/jem.20201045 32725126 PMC7596814

[B26] Dobbs N. Burnaevskiy N. Chen D. Gonugunta V. K. Alto N. M. Yan N. (2015). STING activation by translocation from the ER is associated with infection and autoinflammatory disease. Cell Host Microbe 18 (2), 157–168. 10.1016/j.chom.2015.07.001 26235147 PMC4537353

[B27] Domizio J. D. Gulen M. F. Saidoune F. Thacker V. V. Yatim A. Sharma K. (2022). The cGAS-STING pathway drives type I IFN immunopathology in COVID-19. Nature 603 (7899), 145–151. 10.1038/s41586-022-04421-w 35045565 PMC8891013

[B28] Ergun S. L. Fernandez D. Weiss T. M. Li L. (2019). STING polymer structure reveals mechanisms for activation, hyperactivation, and inhibition. Cell 178 (2), 290–301. 10.1016/j.cell.2019.05.036 31230712

[B29] Fang R. Wang C. Jiang Q. Lv M. Gao P. Yu X. (2017). NEMO-IKKβ are essential for IRF3 and NF-κB activation in the cGAS-STING pathway. J. Immunol. 199 (9), 3222–3233. 10.4049/jimmunol.1700699 28939760

[B30] Feng J. Armillei M. K. Yu A. S. Liang B. T. Runnels L. W. Yue L. (2019). Ca(2+) signaling in cardiac fibroblasts and fibrosis-associated heart diseases. J. Cardiovasc. Dev. Dis. 6 (4), E34. 10.3390/jcdd6040034 PMC6956282 31547577

[B31] Fine A. Goldstein R. H. (1987). The effect of transforming growth factor-beta on cell proliferation and collagen formation by lung fibroblasts. J. Biol. Chem. 262 (8), 3897–3902. 10.1016/s0021-9258(18)61441-3 3493244

[B32] Friedman S. L. Neuschwander-Tetri B. A. Rinella M. Sanyal A. J. (2018). Mechanisms of NAFLD development and therapeutic strategies. Nat. Med. 24 (7), 908–922. 10.1038/s41591-018-0104-9 29967350 PMC6553468

[B33] Galani I. E. Rovina N. Lampropoulou V. Triantafyllia V. Manioudaki M. Pavlos E. (2021). Untuned antiviral immunity in COVID-19 revealed by temporal type I/III interferon patterns and flu comparison. Nat. Immunol. 22 (1), 32–40. 10.1038/s41590-020-00840-x 33277638

[B34] Gall A. Treuting P. Elkon K. B. Loo Y. M. Gale M. Jr. Barber G. N. (2012). Autoimmunity initiates in nonhematopoietic cells and progresses via lymphocytes in an interferon-dependent autoimmune disease. Immunity 36 (1), 120–131. 10.1016/j.immuni.2011.11.018 22284419 PMC3269499

[B35] Gao P. Ascano M. Wu Y. Barchet W. Gaffney B. L. Zillinger T. (2013). Cyclic [G(2', 5')pA(3', 5')p] is the metazoan second messenger produced by DNA-activated cyclic GMP-AMP synthase. Cell 153 (5), 1094–1107. 10.1016/j.cell.2013.04.046 23647843 PMC4382009

[B36] Gao D. Li T. Li X. D. Chen X. Li Q. Z. Wight-Carter M. (2015). Activation of cyclic GMP-AMP synthase by self-DNA causes autoimmune diseases. Proc. Natl. Acad. Sci. U. S. A. 112 (42), E5699–E5705. 10.1073/pnas.1516465112 26371324 PMC4620884

[B37] Gkirtzimanaki K. Kabrani E. Nikoleri D. Polyzos A. Blanas A. Sidiropoulos P. (2018). IFNα impairs autophagic degradation of mtDNA promoting autoreactivity of SLE monocytes in a STING-dependent fashion. Cell Rep. 25 (4), 921–933.e5. 10.1016/j.celrep.2018.09.001 30355498 PMC6218203

[B38] Glück S. Guey B. Gulen M. F. Wolter K. Kang T. W. Schmacke N. A. (2017). Innate immune sensing of cytosolic chromatin fragments through cGAS promotes senescence. Nat. Cell Biol. 19 (9), 1061–1070. 10.1038/ncb3586 28759028 PMC5826565

[B39] Goh G. B. Pagadala M. R. Dasarathy J. Unalp-Arida A. Sargent R. Hawkins C. (2015). Renin-angiotensin system and fibrosis in non-alcoholic fatty liver disease. Liver Int. 35 (3), 979–985. 10.1111/liv.12611 24905085

[B40] Gui X. Yang H. Li T. Tan X. Shi P. Li M. (2019). Autophagy induction via STING trafficking is a primordial function of the cGAS pathway. Nature 567 (7747), 262–266. 10.1038/s41586-019-1006-9 30842662 PMC9417302

[B41] Haag S. M. Gulen M. F. Reymond L. Gibelin A. Abrami L. Decout A. (2018). Targeting STING with covalent small-molecule inhibitors. Nature 559 (7713), 269–273. 10.1038/s41586-018-0287-8 29973723

[B42] Han B. Wang X. Wu P. Jiang H. Yang Q. Li S. (2021). Pulmonary inflammatory and fibrogenic response induced by graphitized multi-walled carbon nanotube involved in cGAS-STING signaling pathway. J. Hazard. Mater. 417, 125984. 10.1016/j.jhazmat.2021.125984 34020360

[B43] Hansen A. L. Buchan G. J. Rühl M. Mukai K. Salvatore S. R. Ogawa E. (2018). Nitro-fatty acids are formed in response to virus infection and are potent inhibitors of STING palmitoylation and signaling. Proc. Natl. Acad. Sci. U. S. A. 115 (33), E7768–e7775. 10.1073/pnas.1806239115 30061387 PMC6099880

[B44] Hao J. Huang X. Guan J. Feng J. Li D. Cao S. (2022). Shenkang injection protects against renal fibrosis by reducing perforin expression through the STING/TBK1/IRF3 signaling pathways in natural killer cells. Phytomedicine. 104, 154206. 10.1016/j.phymed.2022.154206 35724525

[B45] He X. Wang Y. Fan X. Lei N. Tian Y. Zhang D. (2020). A schistosome miRNA promotes host hepatic fibrosis by targeting transforming growth factor beta receptor III. J. Hepatol. 72 (3), 519–527. 10.1016/j.jhep.2019.10.029 31738999

[B46] Heldin C. H. Moustakas A. (2016). Signaling receptors for TGF-β family members. Cold Spring Harb. Perspect. Biol. 8 (8), a022053. 10.1101/cshperspect.a022053 27481709 PMC4968163

[B47] Henderson N. C. Rieder F. Wynn T. A. (2020). Fibrosis: from mechanisms to medicines. Nature 587 (7835), 555–566. 10.1038/s41586-020-2938-9 33239795 PMC8034822

[B48] Hinkle J. T. Patel J. Panicker N. Karuppagounder S. S. Biswas D. Belingon B. (2022). STING mediates neurodegeneration and neuroinflammation in nigrostriatal α-synucleinopathy. Proc. Natl. Acad. Sci. U. S. A. 119 (15), e2118819119. 10.1073/pnas.2118819119 35394877 PMC9169780

[B49] Hong Z. Mei J. Li C. Bai G. Maimaiti M. Hu H. (2021). STING inhibitors target the cyclic dinucleotide binding pocket. Proc. Natl. Acad. Sci. U. S. A. 118 (24), e2105465118. 10.1073/pnas.2105465118 34099558 PMC8214703

[B50] Hopfner K. P. Hornung V. (2020). Molecular mechanisms and cellular functions of cGAS-STING signalling. Nat. Rev. Mol. Cell Biol. 21 (9), 501–521. 10.1038/s41580-020-0244-x 32424334

[B51] Horowitz J. C. Thannickal V. J. (2019). Mechanisms for the resolution of organ fibrosis. Physiol. (Bethesda) 34 (1), 43–55. 10.1152/physiol.00033.2018 PMC6383633 30540232

[B52] Hou Y. Wei Y. Lautrup S. Yang B. Wang Y. Cordonnier S. (2021). NAD(+) supplementation reduces neuroinflammation and cell senescence in a transgenic mouse model of Alzheimer's disease via cGAS-STING. Proc. Natl. Acad. Sci. U. S. A. 118 (37), E1876–E1885. 10.1073/pnas.1718819115 PMC8449423 34497121

[B53] Hu H. H. Chen D. Q. Wang Y. N. Feng Y. L. Cao G. Vaziri N. D. (2018). New insights into TGF-β/Smad signaling in tissue fibrosis. Chem. Biol. Interact. 292, 76–83. 10.1016/j.cbi.2018.07.008 30017632

[B54] Hu D. Cui Y. X. Wu M. Y. Li L. Su L. N. Lian Z. (2020). Cytosolic DNA sensor cGAS plays an essential pathogenetic role in pressure overload-induced heart failure. Am. J. Physiol. Heart Circ. Physiol. 318 (6), H1525–H1537. 10.1152/ajpheart.00097.2020 32383996

[B55] Hu H. H. Cao G. Wu X. Q. Vaziri N. D. Zhao Y. Y. (2020). Wnt signaling pathway in aging-related tissue fibrosis and therapies. Ageing Res. Rev. 60, 101063. 10.1016/j.arr.2020.101063 32272170

[B56] Hu S. Gao Y. Gao R. Wang Y. Qu Y. Yang J. (2022). The selective STING inhibitor H-151 preserves myocardial function and ameliorates cardiac fibrosis in murine myocardial infarction. Int. Immunopharmacol. 107, 108658. 10.1016/j.intimp.2022.108658 35278833

[B57] Iracheta-Vellve A. Petrasek J. Gyongyosi B. Satishchandran A. Lowe P. Kodys K. (2016). Endoplasmic reticulum stress-induced hepatocellular death pathways mediate liver injury and fibrosis via stimulator of interferon genes. J. Biol. Chem. 291 (52), 26794–26805. 10.1074/jbc.M116.736991 27810900 PMC5207187

[B58] Ishikawa H. Barber G. N. (2008). STING is an endoplasmic reticulum adaptor that facilitates innate immune signalling. Nature 455 (7213), 674–678. 10.1038/nature07317 18724357 PMC2804933

[B59] Ishikawa H. Ma Z. Barber G. N. (2009). STING regulates intracellular DNA-mediated, type I interferon-dependent innate immunity. Nature 461 (7265), 788–792. 10.1038/nature08476 19776740 PMC4664154

[B60] Jack C. R. Jr. Bennett D. A. Blennow K. Carrillo M. C. Dunn B. Haeberlein S. B. (2018). NIA-AA Research Framework: Toward a biological definition of Alzheimer's disease. Alzheimers Dement. 14 (4), 535–562. 10.1016/j.jalz.2018.02.018 29653606 PMC5958625

[B61] Jiang M. Chen P. Wang L. Li W. Chen B. Liu Y. (2020). cGAS-STING, an important pathway in cancer immunotherapy. J. Hematol. Oncol. 13 (1), 81. 10.1186/s13045-020-00916-z 32571374 PMC7310007

[B62] Jin L. Waterman P. M. Jonscher K. R. Short C. M. Reisdorph N. A. Cambier J. C. (2008). MPYS, a novel membrane tetraspanner, is associated with major histocompatibility complex class II and mediates transduction of apoptotic signals. Mol. Cell. Biol. 28 (16), 5014–5026. 10.1128/mcb.00640-08 18559423 PMC2519703

[B63] Kato Y. Park J. Takamatsu H. Konaka H. Aoki W. Aburaya S. (2018). Apoptosis-derived membrane vesicles drive the cGAS-STING pathway and enhance type I IFN production in systemic lupus erythematosus. Ann. Rheum. Dis. 77 (10), 1507–1515. 10.1136/annrheumdis-2018-212988 29945921 PMC6161667

[B64] Kisseleva T. Brenner D. (2021). Molecular and cellular mechanisms of liver fibrosis and its regression. Nat. Rev. Gastroenterol. Hepatol. 18 (3), 151–166. 10.1038/s41575-020-00372-7 33128017

[B65] Konno H. Konno K. Barber G. N. (2013). Cyclic dinucleotides trigger ULK1 (ATG1) phosphorylation of STING to prevent sustained innate immune signaling. Cell 155 (3), 688–698. 10.1016/j.cell.2013.09.049 24119841 PMC3881181

[B66] Lee J. S. Shin E. C. (2020). The type I interferon response in COVID-19: implications for treatment. Nat. Rev. Immunol. 20 (10), 585–586. 10.1038/s41577-020-00429-3 32788708 PMC8824445

[B67] Lee J. S. Park S. Jeong H. W. Ahn J. Y. Choi S. J. Lee H. (2020). Immunophenotyping of COVID-19 and influenza highlights the role of type I interferons in development of severe COVID-19. Sci. Immunol. 5 (49), eabd1554. 10.1126/sciimmunol.abd1554 32651212 PMC7402635

[B68] Lee-Kirsch M. A. Gong M. Chowdhury D. Senenko L. Engel K. Lee Y. A. (2007). Mutations in the gene encoding the 3'-5' DNA exonuclease TREX1 are associated with systemic lupus erythematosus. Nat. Genet. 39 (9), 1065–1067. 10.1038/ng2091 17660818

[B69] Lepelley A. Martin-Niclós M. J. Le Bihan M. Marsh J. A. Uggenti C. Rice G. I. (2020). Mutations in COPA lead to abnormal trafficking of STING to the Golgi and interferon signaling. J. Exp. Med. 217 (11), e20200600. 10.1084/jem.20200600 32725128 PMC7596811

[B70] Li S. Hong Z. Wang Z. Li F. Mei J. Huang L. (2018). The cyclopeptide Astin C specifically inhibits the innate immune CDN sensor STING. Cell Rep. 25 (12), 3405–3421. 10.1016/j.celrep.2018.11.097 30566866

[B71] Li A. Yi M. Qin S. Song Y. Chu Q. Wu K. (2019). Activating cGAS-STING pathway for the optimal effect of cancer immunotherapy. J. Hematol. Oncol. 12 (1), 35. 10.1186/s13045-019-0721-x 30935414 PMC6444510

[B72] Li N. Zhou H. Wu H. Wu Q. Duan M. Deng W. (2019). STING-IRF3 contributes to lipopolysaccharide-induced cardiac dysfunction, inflammation, apoptosis and pyroptosis by activating NLRP3. Redox Biol. 24, 101215. 10.1016/j.redox.2019.101215 31121492 PMC6529775

[B73] Liang L. Shen Y. Hu Y. Liu H. Cao J. (2022). cGAS exacerbates Schistosoma japonicum infection in a STING-type I IFN-dependent and independent manner. PLoS Pathog. 18 (2), e1010233. 10.1371/journal.ppat.1010233 35108342 PMC8809611

[B74] Liu Y. Jesus A. A. Marrero B. Yang D. Ramsey S. E. Sanchez G. A. M. (2014). Activated STING in a vascular and pulmonary syndrome. N. Engl. J. Med. 371 (6), 507–518. 10.1056/NEJMoa1312625 25029335 PMC4174543

[B75] Liu S. Cai X. Wu J. Cong Q. Chen X. Li T. (2015). Phosphorylation of innate immune adaptor proteins MAVS, STING, and TRIF induces IRF3 activation. Science 347 (6227), aaa2630. 10.1126/science.aaa2630 25636800

[B76] Liu Z. Huang X. R. Chen H. Y. Fung E. Liu J. Lan H. Y. (2017). Deletion of angiotensin-converting enzyme-2 promotes hypertensive nephropathy by targeting Smad7 for ubiquitin degradation. Hypertension 70 (4), 822–830. 10.1161/hypertensionaha.117.09600 28808068

[B77] Liu C. Tang J. Luo W. Liu S. Sun X. Hong W. (2022). DNA from macrophages induces fibrosis and vasculopathy through POLR3A/STING/type I interferon axis in systemic sclerosis. Rheumatol. Oxf., keac324. 10.1093/rheumatology/keac324 35686918

[B78] Liu K. Zhao X. Guo M. Zhu J. Li D. Ding J. (2022). Microcystin-leucine arginine (MC-LR) induces mouse ovarian inflammation by promoting granulosa cells to produce inflammatory cytokine via activation of cGAS-STING signaling. Toxicol. Lett. 358, 6–16. 10.1016/j.toxlet.2022.01.003 35032610

[B79] Liu X. Wei L. Xu F. Zhao F. Huang Y. Fan Z. (2022c). SARS-CoV-2 spike protein-induced cell fusion activates the cGAS-STING pathway and the interferon response. Sci. Signal. 15 (729), eabg8744. 10.1126/scisignal.abg8744 35412852

[B80] Luecke S. Holleufer A. Christensen M. H. Jønsson K. L. Boni G. A. Sørensen L. K. (2017). cGAS is activated by DNA in a length-dependent manner. EMBO Rep. 18 (10), 1707–1715. 10.15252/embr.201744017 28801534 PMC5623850

[B81] Luo X. Li H. Ma L. Zhou J. Guo X. Woo S. L. (2018). Expression of STING is increased in liver tissues from patients with NAFLD and promotes macrophage-mediated hepatic inflammation and fibrosis in mice. Gastroenterology 155 (6), 1971–1984. 10.1053/j.gastro.2018.09.010 30213555 PMC6279491

[B82] Luo Z. Ji Y. Zhang D. Gao H. Jin Z. Yang M. (2022). Microbial DNA enrichment promotes liver steatosis and fibrosis in the course of non-alcoholic steatohepatitis. Acta Physiol. 235 (3), e13827. 10.1111/apha.13827 PMC9335517 35500155

[B83] Mack M. Yanagita M. (2015). Origin of myofibroblasts and cellular events triggering fibrosis. Kidney Int. 87 (2), 297–307. 10.1038/ki.2014.287 25162398

[B84] Mack M. (2018). Inflammation and fibrosis. Matrix Biol. 68-69, 106–121. 10.1016/j.matbio.2017.11.010 29196207

[B85] Mackenzie K. J. Carroll P. Lettice L. Tarnauskaitė Ž. Reddy K. Dix F. (2016). Ribonuclease H2 mutations induce a cGAS/STING-dependent innate immune response. Embo J. 35 (8), 831–844. 10.15252/embj.201593339 26903602 PMC4855687

[B86] McCauley M. E. O'Rourke J. G. Yáñez A. Markman J. L. Ho R. Wang X. (2020). C9orf72 in myeloid cells suppresses STING-induced inflammation. Nature 585 (7823), 96–101. 10.1038/s41586-020-2625-x 32814898 PMC7484469

[B87] McDonald L. T. (2021). Healing after COVID-19: are survivors at risk for pulmonary fibrosis? Am. J. Physiol. Lung Cell. Mol. Physiol. 320 (2), L257–l265. 10.1152/ajplung.00238.2020 33355522 PMC7900916

[B88] Mejzini R. Flynn L. L. Pitout I. L. Fletcher S. Wilton S. D. Akkari P. A. (2019). ALS genetics, mechanisms, and therapeutics: Where are we now? Front. Neurosci. 13, 1310. 10.3389/fnins.2019.01310 31866818 PMC6909825

[B89] Meng X. M. Nikolic-Paterson D. J. Lan H. Y. (2016). TGF-β: the master regulator of fibrosis. Nat. Rev. Nephrol. 12 (6), 325–338. 10.1038/nrneph.2016.48 27108839

[B90] Meric-Bernstam F. Sweis R. F. Hodi F. S. Messersmith W. A. Andtbacka R. H. I. Ingham M. (2022). Phase I dose-escalation trial of MIW815 (ADU-S100), an intratumoral STING agonist, in patients with advanced/metastatic solid tumors or lymphomas. Clin. Cancer Res. 28 (4), 677–688. 10.1158/1078-0432.Ccr-21-1963 34716197

[B91] Mezzano S. A. Ruiz-Ortega M. Egido J. (2001). Angiotensin II and renal fibrosis. Hypertension 38, 635–638. 10.1161/hy09t1.094234 11566946

[B92] Montero P. Milara J. Roger I. Cortijo J. (2021). Role of JAK/STAT in interstitial lung diseases; molecular and cellular mechanisms. Int. J. Mol. Sci. 22 (12), 6211. 10.3390/ijms22126211 34207510 PMC8226626

[B93] Morris R. Kershaw N. J. Babon J. J. (2018). The molecular details of cytokine signaling via the JAK/STAT pathway. Protein Sci. 27 (12), 1984–2009. 10.1002/pro.3519 30267440 PMC6237706

[B94] Mukai K. Konno H. Akiba T. Uemura T. Waguri S. Kobayashi T. (2016). Activation of STING requires palmitoylation at the Golgi. Nat. Commun. 7, 11932. 10.1038/ncomms11932 27324217 PMC4919521

[B95] Murayama G. Chiba A. Kuga T. Makiyama A. Yamaji K. Tamura N. (2020). Inhibition of mTOR suppresses IFNα production and the STING pathway in monocytes from systemic lupus erythematosus patients. Rheumatol. Oxf. 59 (10), 2992–3002. 10.1093/rheumatology/keaa060 32160289

[B96] Murthy A. M. V. Robinson N. Kumar S. (2020). Crosstalk between cGAS-STING signaling and cell death. Cell Death Differ. 27 (11), 2989–3003. 10.1038/s41418-020-00624-8 32948836 PMC7560597

[B97] Nakamura T. Sato T. Endo R. Sasaki S. Takahashi N. Sato Y. (2021). STING agonist loaded lipid nanoparticles overcome anti-PD-1 resistance in melanoma lung metastasis via NK cell activation. J. Immunother. Cancer 9 (7), e002852. 10.1136/jitc-2021-002852 34215690 PMC8256839

[B98] Nascimento M. Gombault A. Lacerda-Queiroz N. Panek C. Savigny F. Sbeity M. (2019). Self-DNA release and STING-dependent sensing drives inflammation to cigarette smoke in mice. Sci. Rep. 9 (1), 14848. 10.1038/s41598-019-51427-y 31619733 PMC6795997

[B99] Neufeldt C. J. Cerikan B. Cortese M. Frankish J. Lee J. Y. Plociennikowska A. (2022). SARS-CoV-2 infection induces a pro-inflammatory cytokine response through cGAS-STING and NF-κB. Commun. Biol. 5 (1), 45. 10.1038/s42003-021-02983-5 35022513 PMC8755718

[B100] Nguyen H. Q. To N. H. Zadigue P. Kerbrat S. De La Taille A. Le Gouvello S. (2018). Ionizing radiation-induced cellular senescence promotes tissue fibrosis after radiotherapy. A review. Crit. Rev. Oncol. Hematol. 129, 13–26. 10.1016/j.critrevonc.2018.06.012 30097231

[B101] Pierantonelli I. Svegliati-Baroni G. (2019). Nonalcoholic fatty liver disease: Basic pathogenetic mechanisms in the progression from NAFLD to NASH. Transplantation 103 (1), e1–e13. 10.1097/tp.0000000000002480 30300287

[B102] Pober J. S. Sessa W. C. (2007). Evolving functions of endothelial cells in inflammation. Nat. Rev. Immunol. 7 (10), 803–815. 10.1038/nri2171 17893694

[B103] Prabakaran T. Troldborg A. Kumpunya S. Alee I. Marinković E. Windross S. J. (2021). A STING antagonist modulating the interaction with STIM1 blocks ER-to-Golgi trafficking and inhibits lupus pathology. EBioMedicine 66, 103314. 10.1016/j.ebiom.2021.103314 33813142 PMC8047499

[B104] Qin W. Cao L. Massey I. Y. (2021). Role of PI3K/Akt signaling pathway in cardiac fibrosis. Mol. Cell. Biochem. 476 (11), 4045–4059. 10.1007/s11010-021-04219-w 34244974

[B105] Rech L. Abdellatif M. Pöttler M. Stangl V. Mabotuwana N. Hardy S. (2022). Small molecule STING inhibition improves myocardial infarction remodeling. Life Sci. 291, 120263. 10.1016/j.lfs.2021.120263 34971697

[B106] Ren H. Ma C. Peng H. Zhang B. Zhou L. Su Y. (2021). Micronucleus production, activation of DNA damage response and cGAS-STING signaling in syncytia induced by SARS-CoV-2 infection. Biol. Direct 16 (1), 20. 10.1186/s13062-021-00305-7 34674770 PMC8530504

[B107] Renton A. E. Majounie E. Waite A. Simón-Sánchez J. Rollinson S. Gibbs J. R. (2011). A hexanucleotide repeat expansion in C9ORF72 is the cause of chromosome 9p21-linked ALS-FTD. Neuron 72 (2), 257–268. 10.1016/j.neuron.2011.09.010 21944779 PMC3200438

[B108] Rice G. Newman W. G. Dean J. Patrick T. Parmar R. Flintoff K. (2007). Heterozygous mutations in TREX1 cause familial chilblain lupus and dominant Aicardi-Goutieres syndrome. Am. J. Hum. Genet. 80 (4), 811–815. 10.1086/513443 17357087 PMC1852703

[B109] Rice G. I. Rodero M. P. Crow Y. J. (2015). Human disease phenotypes associated with mutations in TREX1. J. Clin. Immunol. 35 (3), 235–243. 10.1007/s10875-015-0147-3 25731743

[B110] Rockey D. C. Bell P. D. Hill J. A. (2015). Fibrosis--a common pathway to organ injury and failure. N. Engl. J. Med. 372 (12), 1138–1149. 10.1056/NEJMra1300575 25785971

[B111] Romagnani P. Remuzzi G. Glassock R. Levin A. Jager K. J. Tonelli M. (2017). Chronic kidney disease. Nat. Rev. Dis. Prim. 3, 17088. 10.1038/nrdp.2017.88 29168475

[B112] Scheltens P. De Strooper B. Kivipelto M. Holstege H. Chételat G. Teunissen C. E. (2021). Alzheimer's disease. Lancet 397 (10284), 1577–1590. 10.1016/s0140-6736(20)32205-4 33667416 PMC8354300

[B113] Schuster S. Cabrera D. Arrese M. Feldstein A. E. (2018). Triggering and resolution of inflammation in NASH. Nat. Rev. Gastroenterol. Hepatol. 15 (6), 349–364. 10.1038/s41575-018-0009-6 29740166

[B114] Seo S. U. Jeong J. H. Baek B. S. Choi J. M. Choi Y. S. Ko H. J. (2021). Bleomycin-induced lung injury increases resistance to influenza virus infection in a type I interferon-dependent manner. Front. Immunol. 12, 697162. 10.3389/fimmu.2021.697162 34484196 PMC8416411

[B115] Shang G. Zhang C. Chen Z. J. Bai X. C. Zhang X. (2019). Cryo-EM structures of STING reveal its mechanism of activation by cyclic GMP-AMP. Nature 567 (7748), 389–393. 10.1038/s41586-019-0998-5 30842659 PMC6859894

[B116] Shen R. Yang K. Cheng X. Guo C. Xing X. Sun H. (2022). Accumulation of polystyrene microplastics induces liver fibrosis by activating cGAS/STING pathway. Environ. Pollut. 300, 118986. 10.1016/j.envpol.2022.118986 35167931

[B117] Shi W. Hao J. Wu Y. Liu C. Shimizu K. Li R. (2022). Protective effects of heterophyllin B against bleomycin-induced pulmonary fibrosis in mice via AMPK activation. Eur. J. Pharmacol. 921, 174825. 10.1016/j.ejphar.2022.174825 35283110

[B118] Shmuel-Galia L. Humphries F. Lei X. Ceglia S. Wilson R. Jiang Z. (2021). Dysbiosis exacerbates colitis by promoting ubiquitination and accumulation of the innate immune adaptor STING in myeloid cells. Immunity 54 (6), 1137–1153.e8. 10.1016/j.immuni.2021.05.008 34051146 PMC8237382

[B119] Siu T. Altman M. D. Baltus G. A. Childers M. Ellis J. M. Gunaydin H. (2019). Discovery of a novel cGAMP competitive ligand of the inactive form of STING. ACS Med. Chem. Lett. 10 (1), 92–97. 10.1021/acsmedchemlett.8b00466 30655953 PMC6331172

[B120] Sun W. Li Y. Chen L. Chen H. You F. Zhou X. (2009). ERIS, an endoplasmic reticulum IFN stimulator, activates innate immune signaling through dimerization. Proc. Natl. Acad. Sci. U. S. A. 106 (21), 8653–8658. 10.1073/pnas.0900850106 19433799 PMC2689030

[B121] Sun L. Wu J. Du F. Chen X. Chen Z. J. (2013). Cyclic GMP-AMP synthase is a cytosolic DNA sensor that activates the type I interferon pathway. Science 339 (6121), 786–791. 10.1126/science.1232458 23258413 PMC3863629

[B122] Sun S. C. Han R. Hou S. S. Yi H. Q. Chi S. J. Zhang A. H. (2020a). Juglanin alleviates bleomycin-induced lung injury by suppressing inflammation and fibrosis via targeting sting signaling. Biomed. Pharmacother. 127, 110119. 10.1016/j.biopha.2020.110119 32276127

[B123] Sun Z. Yang Z. Wang M. Huang C. Ren Y. Zhang W. (2020b). Paraquat induces pulmonary fibrosis through Wnt/β-catenin signaling pathway and myofibroblast differentiation. Toxicol. Lett. 333, 170–183. 10.1016/j.toxlet.2020.08.004 32795487

[B124] Takahashi A. Loo T. M. Okada R. Kamachi F. Watanabe Y. Wakita M. (2018). Downregulation of cytoplasmic DNases is implicated in cytoplasmic DNA accumulation and SASP in senescent cells. Nat. Commun. 9 (1), 1249. 10.1038/s41467-018-03555-8 29593264 PMC5871854

[B125] Tan H. Y. Yong Y. K. Xue Y. C. Liu H. Furihata T. Shankar E. M. (2022). cGAS and DDX41-STING mediated intrinsic immunity spreads intercellularly to promote neuroinflammation in SOD1 ALS model. iScience 25 (6), 104404. 10.1016/j.isci.2022.104404 35712074 PMC9194172

[B126] Thim-Uam A. Prabakaran T. Tansakul M. Makjaroen J. Wongkongkathep P. Chantaravisoot N. (2020). STING mediates lupus via the activation of conventional dendritic cell maturation and plasmacytoid dendritic cell differentiation. iScience 23 (9), 101530. 10.1016/j.isci.2020.101530 33083760 PMC7502826

[B127] Tsuchida T. Friedman S. L. (2017). Mechanisms of hepatic stellate cell activation. Nat. Rev. Gastroenterol. Hepatol. 14 (7), 397–411. 10.1038/nrgastro.2017.38 28487545

[B128] Ung C. Y. Onoufriadis A. Parsons M. McGrath J. A. Shaw T. J. (2021). Metabolic perturbations in fibrosis disease. Int. J. Biochem. Cell Biol. 139, 106073. 10.1016/j.biocel.2021.106073 34461262

[B129] Villiger P. M. Kusari A. B. ten Dijke P. Lotz M. (1993). IL-1 beta and IL-6 selectively induce transforming growth factor-beta isoforms in human articular chondrocytes. J. Immunol. 151 (6), 3337–3344. 8376781

[B130] Volpi S. Tsui J. Mariani M. Pastorino C. Caorsi R. Sacco O. (2018). Type I interferon pathway activation in COPA syndrome. Clin. Immunol. 187, 33–36. 10.1016/j.clim.2017.10.001 29030294

[B131] Wang J. Dai M. Cui Y. Hou G. Deng J. Gao X. (2018). Association of abnormal elevations in IFIT3 with overactive cyclic GMP-AMP synthase/stimulator of interferon genes signaling in human systemic lupus erythematosus monocytes. Arthritis Rheumatol. 70 (12), 2036–2045. 10.1002/art.40576 29806091

[B132] Wang N. Zhan Y. Zhu L. Hou Z. Liu F. Song P. (2020). Retrospective multicenter cohort study shows early interferon therapy is associated with favorable clinical responses in COVID-19 patients. Cell Host Microbe 28 (3), 455–464.e2. 10.1016/j.chom.2020.07.005 32707096 PMC7368656

[B133] Wang X. Rao H. Zhao J. Wee A. Li X. Fei R. (2020). STING expression in monocyte-derived macrophages is associated with the progression of liver inflammation and fibrosis in patients with nonalcoholic fatty liver disease. Lab. Invest. 100 (4), 542–552. 10.1038/s41374-019-0342-6 31745210

[B134] Wang Y. Luo J. Alu A. Han X. Wei Y. Wei X. (2020). cGAS-STING pathway in cancer biotherapy. Mol. Cancer 19 (1), 136. 10.1186/s12943-020-01247-w 32887628 PMC7472700

[B135] Wang K. Donnelly C. R. Jiang C. Liao Y. Luo X. Tao X. (2021). STING suppresses bone cancer pain via immune and neuronal modulation. Nat. Commun. 12 (1), 4558. 10.1038/s41467-021-24867-2 34315904 PMC8316360

[B136] Wang Q. Bergholz J. S. Ding L. Lin Z. Kabraji S. K. Hughes M. E. (2022). STING agonism reprograms tumor-associated macrophages and overcomes resistance to PARP inhibition in BRCA1-deficient models of breast cancer. Nat. Commun. 13 (1), 3022. 10.1038/s41467-022-30568-1 35641483 PMC9156717

[B137] Willemsen J. Neuhoff M. T. Hoyler T. Noir E. Tessier C. Sarret S. (2021). TNF leads to mtDNA release and cGAS/STING-dependent interferon responses that support inflammatory arthritis. Cell Rep. 37 (6), 109977. 10.1016/j.celrep.2021.109977 34758308

[B138] Wu J. Sun L. Chen X. Du F. Shi H. Chen C. (2013). Cyclic GMP-AMP is an endogenous second messenger in innate immune signaling by cytosolic DNA. Science 339 (6121), 826–830. 10.1126/science.1229963 23258412 PMC3855410

[B139] Wu J. Raman A. Coffey N. J. Sheng X. Wahba J. Seasock M. J. (2021). The key role of NLRP3 and STING in APOL1-associated podocytopathy. J. Clin. Invest. 131 (20), e136329. 10.1172/jci136329 34651582 PMC8516463

[B140] Xiong Y. Tang Y. D. Zheng C. (2021). The crosstalk between the caspase family and the cGAS‒STING signaling pathway. J. Mol. Cell Biol. 13 (10), 739–747. 10.1093/jmcb/mjab071 34718659 PMC8718194

[B141] Yang M. Fan Q. Hei T. K. Chen G. Cao W. Meng G. (2022a). Single-cell transcriptome analysis of radiation pneumonitis mice. Antioxidants (Basel) 11 (8), 1457. 10.3390/antiox11081457 35892659 PMC9331247

[B142] Yang M. Ma Y. X. Zhi Y. Wang H. B. Zhao L. Wang P. S. (2022b). Inhibitors of IFN gene stimulators (STING) improve intestinal ischemia-reperfusion-induced acute lung injury by activating AMPK signaling. Eur. J. Med. Res. 27 (1), 79. 10.1186/s40001-022-00703-1 35642042 PMC9153160

[B143] Yong H. Wang S. Song F. (2021). Activation of cGAS/STING pathway upon TDP-43-mediated mitochondrial injury may be involved in the pathogenesis of liver fibrosis. Liver Int. 41 (8), 1969–1971. 10.1111/liv.14895 33830629

[B144] Yu Y. Liu Y. An W. Song J. Zhang Y. Zhao X. (2019). STING-mediated inflammation in Kupffer cells contributes to progression of nonalcoholic steatohepatitis. J. Clin. Invest. 129 (2), 546–555. 10.1172/jci121842 30561388 PMC6355218

[B145] Yu C. H. Davidson S. Harapas C. R. Hilton J. B. Mlodzianoski M. J. Laohamonthonkul P. (2020). TDP-43 triggers mitochondrial DNA release via mPTP to activate cGAS/STING in ALS. Cell 183 (3), 636–649.e18. 10.1016/j.cell.2020.09.020 33031745 PMC7599077

[B146] Zhang M. Zhang S. (2020). T cells in fibrosis and fibrotic diseases. Front. Immunol. 11, 1142. 10.3389/fimmu.2020.01142 32676074 PMC7333347

[B147] Zhang X. Shi H. Wu J. Zhang X. Sun L. Chen C. (2013). Cyclic GMP-AMP containing mixed phosphodiester linkages is an endogenous high-affinity ligand for STING. Mol. Cell 51 (2), 226–235. 10.1016/j.molcel.2013.05.022 23747010 PMC3808999

[B148] Zhang C. Shang G. Gui X. Zhang X. Bai X. C. Chen Z. J. (2019). Structural basis of STING binding with and phosphorylation by TBK1. Nature 567 (7748), 394–398. 10.1038/s41586-019-1000-2 30842653 PMC6862768

[B149] Zhang Y. Chen W. Wang Y. (2020). STING is an essential regulator of heart inflammation and fibrosis in mice with pathological cardiac hypertrophy via endoplasmic reticulum (ER) stress. Biomed. Pharmacother. 125, 110022. 10.1016/j.biopha.2020.110022 32106379

[B150] Zhang Y. Jin D. Kang X. Zhou R. Sun Y. Lian F. (2021). Signaling pathways involved in diabetic renal fibrosis. Front. Cell Dev. Biol. 9, 696542. 10.3389/fcell.2021.696542 34327204 PMC8314387

[B151] Zhang D. Liu Y. Zhu Y. Zhang Q. Guan H. Liu S. (2022). A non-canonical cGAS-STING-PERK pathway facilitates the translational program critical for senescence and organ fibrosis. Nat. Cell Biol. 24 (5), 766–782. 10.1038/s41556-022-00894-z 35501370

[B152] Zhao B. Du F. Xu P. Shu C. Sankaran B. Bell S. L. (2019). A conserved PLPLRT/SD motif of STING mediates the recruitment and activation of TBK1. Nature 569 (7758), 718–722. 10.1038/s41586-019-1228-x 31118511 PMC6596994

[B153] Zhao H. Wu L. Yan G. Chen Y. Zhou M. Wu Y. (2021). Inflammation and tumor progression: Signaling pathways and targeted intervention. Signal Transduct. Target. Ther. 6 (1), 263. 10.1038/s41392-021-00658-5 34248142 PMC8273155

[B154] Zhong B. Yang Y. Li S. Wang Y. Y. Li Y. Diao F. (2008). The adaptor protein MITA links virus-sensing receptors to IRF3 transcription factor activation. Immunity 29 (4), 538–550. 10.1016/j.immuni.2008.09.003 18818105

[B155] Zhou J. Zhong J. Huang Z. Liao M. Lin S. Chen J. (2018). TAK1 mediates apoptosis via p38 involve in ischemia-induced renal fibrosis. Artif. Cells Nanomed. Biotechnol. 46, 1016–1025. 10.1080/21691401.2018.1442841 29661023

[B156] Zhou Z. Zhang X. Lei X. Xiao X. Jiao T. Ma R. (2021). Sensing of cytoplasmic chromatin by cGAS activates innate immune response in SARS-CoV-2 infection. Signal Transduct. Target. Ther. 6 (1), 382. 10.1038/s41392-021-00800-3 34732709 PMC8564796

