# Supplementary material for: The effect of the cyclic GMP-AMP synthase-stimulator of interferon genes signaling pathway on organ inflammatory injury and fibrosis
Source: Front Pharmacol. 2022 Dec 5;13:1033982. doi: 10.3389/fphar.2022.1033982 (PMC9762484; doi:10.3389/fphar.2022.1033982)
Supplement: Supplementary file 1 [file Table1.pdf]

frontiers

Supplementary Material

1 Supplementary Figures and Tables

1.1 Supplementary Table. Application of agonists and antagonists targeting the cGAS-STING pathway

AGONISTS/ ANTAGONISTS	AGONISTS	AUTHOR	ORGAN/CELL LINE	DISEASE/MODEL	CONCLUSION
AGONISTS	2'3'-cG(s)A(s)MP	Li et al. 2014(Li et al., 2014)	THP-1 cells	/	2'3'-cG(s)A(s)MP is a 2′ 3′ -cGAMP analogue that has affinity for both mouse and human STING. It possesses a significant activating STING activity in human THP-1 and withstands ENPP1's hydrolysis of 2′ 3′ -cGAMP.
	ADU S-100 (MIW815 or ML RR-S2 CDA)	Chelvanambi et al. 2021(Chelvanambi et al., 2021)	CD11c DC	Mouse melanoma model	ADUS-100 retards the growth of melanoma. In addition, it helps to induce the development of VN and TLS, thus enhancing the efficacy of tumor treatment. The above results were similarly seen in ADUS-100-treated CD11c DC.
		Meric-Bernstam et al. 2022(Meric-Bernstam et al., 2022)	Skin, breast, colorectum, etc.	Adult patients with advanced/metastatic solid tumors or lymphoma	Tumor site injections of ADUS-100 were well tolerated and safe, however the clinical results were less successful than predicted from preclinical studies

AGONISTS/ ANTAGONISTS	AGONISTS	AUTHOR	ORGAN/CELL LINE	DISEASE/MODEL	CONCLUSION
					and the total response rate for single agent treatment was not very high.
AGONISTS	MK-1454	Chang et al. 2022(Chang et al., 2022)	THP-1 cells and mBMDCs	MC38 and B16F10 murine tumor models	When administered intra-tumorally to mice, MK-1454 drastically decreased tumor size while promoting the production of pro-inflammatory cytokines. Additionally, when combined with PD-1 blockade therapy, it greatly reduced tumor development, indicating that MK-1454 may be used to increase the effectiveness of PD-1 blockade.
	IACS-8803 and IACS-8779	Ager et al. 2019(Ager et al., 2019)	THP-1 and J774 cells	Mouse melanoma model	IACS-8803 and IACS-8779 stably activated the STING pathway in cell lines cultured in vitro. Additionally, compared to ADUS-100, they have a systemic spectrum of action and show notable anticancer efficacy.
		Ager et al. 2021(Ager et al., 2021)	MDSCs and M2-like macrophages	Mouse PDAC model	IACS-8803 activates the STING pathway much more potently than cGAMP, and it reverses the immunosuppressive state in MDSCs. Furthermore, Myc in both MDSCs and human M2 macrophages was negatively regulated by 8803, thereby participating in tumor suppression. The addition of 8803 to immune

AGONISTS/ ANTAGONISTS	AGONISTS	AUTHOR	ORGAN/CELL LINE	DISEASE/MODEL	CONCLUSION
					checkpoint blockade therapy exhibited a curative effect on PDAC.
AGONISTS	NP-cdGMP	Hanson et al. 2015(Hanson et al., 2015)	Lymph nodes	Mouse melanoma model	Compared to cdGMP, NP-cdGMP has higher concentrations in lymph nodes, enhances immune function in mice, and has significant antitumor activity. In addition, NP-cdGMP does not disseminate systemically and cause widespread inflammation.
	PS-GAMP	Wang et al. 2020(Wang et al., 2020)	Lung	Ferret model	PS-GAMP can access AMs and AECs without destroying the PS layer, thereby activating the cGAS-STING immune pathway and enhancing the defense function of CD8 T cells and DCs. A single dose of PS-GAMP can establish long-term immunity in a short period of time, facilitating early resistance to the influenza virus.
	PC7A	Li et al. 2021(Li et al., 2021)	Tumor tissues of mice and humans	MC38 murine tumor models	Compared with cGAMP, the activation effect of PC7A on STING is more lasting. The combined use of PC7A and cGAMP significantly prolonged the survival time of tumor-bearing mice. An increase in the production of activated STING and downstream cytokines was observed in tissues

AGONISTS/ ANTAGONISTS	AGONISTS	AUTHOR	ORGAN/CELL LINE	DISEASE/MODEL	CONCLUSION
					isolated from human tumors treated with PC7A.
AGONISTS	CMA	Cavlar et al. 2013(Cavlar et al., 2013)	murine macrophages, human PBMCs, and HEK293T cells	/	CMA directly binds to and agonizes mouse STING to exert antiviral effects, but does not respond to human STING.
		Peng et al. 2020(Peng et al., 2020)	BV2 cells	Mouse SAH model	CMA increased microglia activation through the STING pathway in both in vivo and in vitro experiments, which increased the production of pro-inflammatory cytokines and exacerbated neuroinflammatory damage. This demonstrates the detrimental impact of CMA on SAH in aggravating impairment of brain function.
	DMXAA (ASA-404 or vadimezan)	Luo et al. 2018(Luo et al., 2018)	Liver/mice BMDMs and human HSCs	NAFLD mice	Long-term administration of DMXAA increased the amount of inflammatory cytokines and aggravated fat accumulation in the mouse liver. Additionally, DMXAA promoted HSCs activity and the expression of genes linked to fibrosis.

AGONISTS/ ANTAGONISTS	AGONISTS	AUTHOR	ORGAN/CELL LINE	DISEASE/MODEL	CONCLUSION
AGONISTS	DMXAA (ASA-404 or vadimezan)	Lara et al. 2011(Lara et al., 2011)	Lung	NSCLC	In phase III clinical trials, there was no significant difference in the effect of chemotherapy alone or in combination with DMXAA on survival in advanced NSCLC. Therefore, DMXAA did not improve efficacy against advanced NSCLC in humans.
		Jing et al. 2019(Jing et al., 2019)	Pancreas	Mouse pancreatic cancer model	By irritating STING, DMXAA increased survival in mice and reversed the immunosuppressive condition in the tumor microenvironment of pancreatic cancer. Additionally, DMXAA encouraged the activation of dendritic cells and macrophages. The DMXAA therapy also increased the production of cytokines and chemokines that promote inflammation.
		Zhao et al. 2018(Zhao et al., 2018)	Pancreas	Mouse acute pancreatitis model	In mouse pancreatic tissue, DMXAA induced STING activation, which increased IFN-1 production and increased inflammatory cell infiltration. In conclusion, acute pancreatitis is made worse by DMXAA.

AGONISTS/ ANTAGONISTS	AGONISTS	AUTHOR	ORGAN/CELL LINE	DISEASE/MODEL	CONCLUSION
AGONISTS	E7766	Kim et al. 2021(Kim et al., 2021)	THP-1 cells	Mouse model of metastatic tumors in the liver	E7766 is much more active than 2′ 3′ -cGAMP and has affinity for several human STING variants. After intra-tumor injection of E7766, the great majority of mice infected with CT26 cells show appreciable resistance to tumor invasion.
		Huang et al. 2022(Huang et al., 2022)	human PBMCs	Mouse NMIBC model	E7766 efficiently activates the STING pathway to promote the production of IFNs and chemokines, etc. This increases the infiltration of lymphocytes, including NK cells, thereby enhancing anti-tumor immunity in NMIBC mice.
	α-mangostin	Zhang et al. 2018(Zhang et al., 2018)	THP-1 cells and 293T cells	/	α-mangostin is less effective against mouse STING, but it immediately binds and agonizes human STING. Compared to 2′ 3′ -cGAMP, it exhibits a slower beginning of effect but a longer duration of action. Additionally, M2 macrophages treated with α-mangostin displayed M1 macrophage biomarkers, indicating that a phenotypic transition of macrophages may have taken place, favoring resistance to malignancies.

AGONISTS/ ANTAGONISTS	AGONISTS	AUTHOR	ORGAN/CELL LINE	DISEASE/MODEL	CONCLUSION
AGONISTS	BNBC	Zhang et al. 2019(Zhang et al., 2019)	DCs, human PBMCs and HHF cells	/	BNBC specifically agonizes human STING to induce inflammatory responses and IFN production to fight tumors and viruses. In addition, it contributes to the function of dendritic cells and thus participates in immune function.
	MSA-2	Pan et al. 2020(Pan et al., 2020)	Colorectal, Lung	MC38, CT26, B16F10, and LL-2 murine tumor models	MSA-2 is active orally and responsive to STING in both mice and humans. It increases survival in mice with colorectal and lung malignancies and promotes tumor immunity through STING.
	G10	Banerjee et al. 2020(Banerjee et al., 2020)	THP-1 and HEK293T cells	CT26 murine line	Unlike the previous findings of Sali et al.(Sali et al., 2015), this study demonstrated that G10 can directly agonize human-derived STING, which was also confirmed in CT26 cells. However, G10 did not have the ability to activate IRF3 and NF-κB signaling in THP-1 cells.
		Ming et al. 2020(Ming et al., 2020)	3D4/21 and PK-15 cells	Pig models	G10 promoted STING activation and downstream type I interferon responses in porcine cells. In addition, G10 activated NF-κB and NLRP3 inflammasome activity. Notably, NLRP3 inflammasome antagonize the

AGONISTS/ ANTAGONISTS	AGONISTS	AUTHOR	ORGAN/CELL LINE	DISEASE/MODEL	CONCLUSION
					production of IFN-1, reflecting an immune homeostasis.
AGONISTS	diABZI	Ramanjulu et al. 2018(Ramanjulu et al., 2018)	Colon/human PBMCs	CT26 murine tumor models	Strong anticancer activity was seen in mice with colon cancer after intravenously administering diABZI. Additionally, it encouraged the activation of the STING transduction pathway by human PBMCs, as well as the generation of cytokines including TNF-α and IL-6.
		Humphries et al. 2021(Humphries et al., 2021)	Lung/lung epithelial cells	Human ACE2-transgenic mice	Treatment with diABZI prevented SARS-CoV-2 from replicating in lung epithelial cells. Additionally, it increased the infiltration and activation of lymphocytes in the lungs and successfully resisted the negative effects caused by SARS-CoV-2 on mouse lungs.
	SR-717	Chin et al. 2020(Chin et al., 2020)	THP-1 cells and human PBMCs	Mouse melanoma model	By activating STING similarly to how cGAMP does, SR-717 encourages the expression of components involved in the STING pathway. It prevents the growth and spread of mouse melanoma and has an affinity for both mouse and human STING.

AGONISTS/ ANTAGONISTS	AGONISTS	AUTHOR	ORGAN/CELL LINE	DISEASE/MODEL	CONCLUSION
AGONISTS	SHR1032	Song et al. 2022(Song et al., 2022)	THP-1 cells and human PBMCs	MC38 murine tumor models	The effect of interferon production induced by SHR1032 was stable and stronger than that of ADUS-100. It is sensitive to human STING and can activate the downstream reaction of STING in THP-1 cells and human PBMCs. In addition, SHR1032 showed an obvious tumor inhibitory effect in mice.
	CF501	Liu et al. 2022(Liu et al., 2022)	Spleen and lungs/THP-1 cells	Human ACE2-transgenic mice, rabbits, and rhesus macaques	As a vaccine adjuvant, CF501 is more effective than cGAMP at boosting humoral and T-cell immunological activation. CF501 also effectively agonizes STING, as evidenced by a marked increase in inflammatory cytokines and chemokines in THP-1 cells. Additionally, it developed long-lasting viral immunity in mice and NHPs, even for several months, to fend against SARS-CoV-2 reinfection.
ANTAGONISTS	C-178/C-176	Haag et al. 2018(Haag et al., 2018)	Heart	Mice deficient in Trex1	C-178 and C-176 act by inhibiting palmitoylation of STING. The degree of cardiac inflammation in mouse models of autoimmune disease was significantly reduced after treatment with the above drugs. However, these

AGONISTS/ ANTAGONISTS	AGONISTS	AUTHOR	ORGAN/CELL LINE	DISEASE/MODEL	CONCLUSION
					two inhibitors did not show activity against human STING.
ANTAGONISTS	C-178/C-176	Shen et al. 2022(Shen et al., 2022)	Liver/hepatocyte	Mice with liver fibrosis	c-176 inhibits perinuclear translocation of STING and NF-κB translocation to the nucleus. This action lowers the expression of fibronectin, which has been elevated, as well as upregulated pro-inflammatory cytokines.
		Chung et al. 2019(Chung et al., 2019)	Kidney	Mice deficient in TFAM	C-176 attenuated TFAM deficiency-induced kidney inflammation and fibrosis in mice, thereby improving renal function. This was manifested as a reduction in apoptosis, kidney injury, inflammation levels as well as fibrosis.
	H-151	Rech et al. 2022(Rech et al., 2022)	Heart	Mouse MI model	H-151 reduces STING-induced interferon production and levels of cardiac inflammation. In addition, it inhibits cardiac remodeling in reperfused MI, thereby reducing myocardial hypertrophy. H-151 also contributes to the reduction of infarct size and fibrosis marker production in the heart. All of the above effects are manifested as protection of cardiac function.

AGONISTS/ ANTAGONISTS	AGONISTS	AUTHOR	ORGAN/CELL LINE	DISEASE/MODEL	CONCLUSION
	NO2-FAs	Hansen et al. 2018(Hansen et al., 2018)	Fibroblasts from SAVI patients	Mice infected with HSV-2	NO2-FAs exert an inhibitory effect on palmitoylation of STING, thereby reducing the production of type I interferon.
ANTAGONISTS	SN-011	Hong et al. 2021(Hong et al., 2021)	MEFs, HFFs, and BMDMs	Mice deficient in Trex1	SN-011 has a good potency and safety profile, and it competes with CDN to bind STING, thereby inhibiting STING-induced inflammatory responses. Therefore, SN-011 has significant benefits in improving autoimmune diseases and may be an effective drug for the treatment of SAVI.
	ISD017	Prabakaran et al. 2021(Prabakaran et al., 2021)	Spleen and kidney/THP-1 cells and human PBMCs	Mouse lupus model	ISD017 acts by inhibiting the translocation of STING to the Golgi apparatus. The downstream activity of STING was inhibited by ISD017 in both mouse lupus models and samples from lupus patients, thereby reducing the extent of lupus lesions.
	Compound C	Lai et al. 2020(Lai et al., 2020)	THP-1 cells and MEFs	Mice deficient in Trex1	Compound C reduced cGAMP in a cGAS-independent manner, thereby attenuating STING-mediated IFN production and, consequently, ISG levels. This has an ameliorative effect

AGONISTS/ ANTAGONISTS	AGONISTS	AUTHOR	ORGAN/CELL LINE	DISEASE/MODEL	CONCLUSION
					on autoimmune damage in Trex1-deficient mice.
ANTAGONISTS	Astin C	Li et al. 2018(Li et al., 2018)	Heart, stomach, and kidney/BMDMs, MEFs, and human PBMCs	Mice deficient in Trex1	Astin C directly binds to STING and inhibits the link between it and downstream IRF3, thereby impairing the normal function of the cGAS-STING pathway. The consequences of its action include diminished defense against viruses and reduced inflammation in multiple organs.
	Palbociclib	Gao et al. 2022(Gao et al., 2022)	Colon/THP-1, HEK293T cells and human PBMCs	DSS-induced colitis mice and mice deficient in Trex1	Palbociclib acts directly on STING to affect its polymerization and activation, thereby attenuating auto-inflammatory injury in Trex1-deficient mice. In addition, it has similar efficacy to H-151 and has been shown to be protective against DSS-induced colitis in mice.
	Celastrol	Liu et al. 2020(Liu et al., 2020)	Spleen/THP-1 cells and MEFs	Mice deficient in Trex1	Celastrol inhibits IRF3 and NF-κB phosphorylation, thereby suppressing STING-mediated IFN responses and alleviating multi-organ inflammatory damage.

AGONISTS/ ANTAGONISTS	AGONISTS	AUTHOR	ORGAN/CELL LINE	DISEASE/MODEL	CONCLUSION
ANTAGONISTS	X6 (acts on cGAS)	An et al. 2018(An et al., 2018)	Heart, spleen/human PBMCs	Mice deficient in Trex1	X6 inhibits the activity of cGAS, thereby reducing the synthesis of cGAMP. The levels of ISGs were decreased by X6 administration both in mice lacking Trex1 and in the PBMCs of SLE patients. In addition, myocardial inflammation and endocardial fibrosis in the aforementioned mice were also improved by X6.
	Ru.521 (acts on cGAS)	Xu et al. 2020(Xu et al., 2020)	Heart	Mice with sepsis	RU.521 inhibited the activation of cGAS, thereby reducing the inflammatory damage in the hearts of septic mice. In addition, inhibition of cGAS protected mice from cardiac damage caused by oxidative stress and apoptosis, thereby improving cardiac function.
	Aspirin (acts on cGAS)	Dai et al. 2019(Dai et al., 2019)	Heart and spleen/THP-1 cells and human PBMCs	Mice deficient in Trex1	Aspirin can acetylate cGAS to inhibit its activity, thereby blocking the downstream response and leading to a reduction in the production of IFNs and ISGs. In addition, the same results were also seen in PBMCs of AGS patients and in AGS mouse models.

α-SMA, alpha-Smooth muscle actin; AECs, alveolar epithelial cells; AGS, Aicardi-Goutières syndrome; AMs, alveolar macrophages; BMDMs, bone marrow-derived macrophages; CDN, cyclic dinucleotide; cGAMP, cyclic GMP-AMP; cGAS, cyclic GMP-AMP synthase;

Cys91, cysteine 91; cdGMP, cyclic diguanylate monophosphate; DC, dendritic cell; DMXAA, 5,6-dimethylxanthenone-4-acetic acid; DSS, dextran sulphate sodium; ENPP1, ectonucleotide pyrophosphatase/phosphodiesterase 1; ER, endoplasmic reticulum; GMWCNT, graphitized multi-walled carbon nanotubes; HEK293T, human embryonic kidney 293T; HHF, human foreskin fibroblast; HSCs, hepatic stellate cells; IFNs, interferons; IL-6, interleukin-6; IRF3, interferon regulatory factor-3; ISG, interferon-stimulated genes; mBMDC, mouse bone marrow-derived dendritic cells; MDSCs, myeloid-derived suppressor cells; MEFs, mouse embryonic fibroblasts; MI, myocardial infarction; NAFLD, nonalcoholic fatty liver disease; NF-κB, nuclear factor kappa B; NHPs, nonhuman primates; NLRP3, NOD-like receptor family, pyrin domain containing 3; NMIBC, non–muscle invasive bladder cancer; NO2-FAs, nitro-fatty acids; NP, nanoparticle; STING agonists are potent lymph node-targeted vaccine adjuvants NSCLC, non-small cell lung cancer; PBMCs, peripheral blood mononuclear cells; PDAC, pancreatic ductal adenocarcinoma; PK-15, porcine kidney 15; PS, pulmonary surfactant; SAH, subarachnoid hemorrhage; SARS-CoV-2, severe acute respiratory syndrome coronavirus 2; SAVI, stimulator of interferon genes-associated vasculopathy with onset in infancy; SCAP, sterol regulatory element-binding protein (SREBP) cleavage-activating protein; SLE, systemic lupus erythematosus; STIM1, stromal interaction molecule 1; STING, stimulator of interferon genes; SUMO, small ubiquitin-like modifier; TBK1, recombinant TANK binding kinase 1; TFAM, mitochondrial transcription factor A; TGF-β, transforming growth factor-β; TLS, tertiary lymphoid structures; TNF-α, tumor necrotic factor-α; Trex1, human three-prime repair exonuclease 1; VN, vascular normalization;

Ager, C.R., Boda, A., Rajapakshe, K., Lea, S.T., Di Francesco, M.E., Jayaprakash, P., et al. (2021). High potency STING agonists engage unique myeloid pathways to reverse pancreatic cancer immune privilege. *J Immunother Cancer* 9(8). doi: 10.1136/jitc-2021-003246.

Ager, C.R., Zhang, H., Wei, Z., Jones, P., Curran, M.A., and Di Francesco, M.E. (2019). Discovery of IACS-8803 and IACS-8779, potent agonists of stimulator of interferon genes (STING) with robust systemic antitumor efficacy. *Bioorg Med Chem Lett* 29(20)**,** 126640. doi: 10.1016/j.bmcl.2019.126640.

An, J., Woodward, J.J., Lai, W., Minie, M., Sun, X., Tanaka, L., et al. (2018). Inhibition of Cyclic GMP-AMP Synthase Using a Novel Antimalarial Drug Derivative in Trex1-Deficient Mice. *Arthritis Rheumatol* 70(11)**,** 1807-1819. doi: 10.1002/art.40559.

Banerjee, M., Middya, S., Shrivastava, R., Basu, S., Ghosh, R., Pryde, D.C., et al. (2020). G10 is a direct activator of human STING. *PLoS One* 15(9)**,** e0237743. doi: 10.1371/journal.pone.0237743.

Cavlar, T., Deimling, T., Ablasser, A., Hopfner, K.P., and Hornung, V. (2013). Species-specific detection of the antiviral small-molecule compound CMA by STING. *Embo j* 32(10)**,** 1440-1450. doi: 10.1038/emboj.2013.86.

Chang, W., Altman, M.D., Lesburg, C.A., Perera, S.A., Piesvaux, J.A., Schroeder, G.K., et al. (2022). Discovery of MK-1454: A Potent Cyclic Dinucleotide Stimulator of Interferon Genes Agonist for the Treatment of Cancer. *J Med Chem* 65(7)**,** 5675-5689. doi: 10.1021/acs.jmedchem.1c02197.

Chelvanambi, M., Fecek, R.J., Taylor, J.L., and Storkus, W.J. (2021). STING agonist-based treatment promotes vascular normalization and tertiary lymphoid structure formation in the therapeutic melanoma microenvironment. *J Immunother Cancer* 9(2). doi: 10.1136/jitc-2020-001906.

Chin, E.N., Yu, C., Vartabedian, V.F., Jia, Y., Kumar, M., Gamo, A.M., et al. (2020). Antitumor activity of a systemic STING-activating non-nucleotide cGAMP mimetic. *Science* 369(6506)**,** 993-999. doi: 10.1126/science.abb4255.

Chung, K.W., Dhillon, P., Huang, S., Sheng, X., Shrestha, R., Qiu, C., et al. (2019). Mitochondrial Damage and Activation of the STING Pathway Lead to Renal Inflammation and Fibrosis. *Cell Metab* 30(4)**,** 784-799.e785. doi: 10.1016/j.cmet.2019.08.003.

Dai, J., Huang, Y.J., He, X., Zhao, M., Wang, X., Liu, Z.S., et al. (2019). Acetylation Blocks cGAS Activity and Inhibits Self-DNA-Induced Autoimmunity. *Cell* 176(6)**,** 1447-1460.e1414. doi: 10.1016/j.cell.2019.01.016.

Gao, J., Zheng, M., Wu, X., Zhang, H., Su, H., Dang, Y., et al. (2022). CDK inhibitor Palbociclib targets STING to alleviate autoinflammation. *EMBO Rep* 23(6)**,** e53932. doi: 10.15252/embr.202153932.

Haag, S.M., Gulen, M.F., Reymond, L., Gibelin, A., Abrami, L., Decout, A., et al. (2018). Targeting STING with covalent small-molecule inhibitors. *Nature* 559(7713)**,** 269-273. doi: 10.1038/s41586-018-0287-8.

Hansen, A.L., Buchan, G.J., Rühl, M., Mukai, K., Salvatore, S.R., Ogawa, E., et al. (2018). Nitro-fatty acids are formed in response to virus infection and are potent inhibitors of STING palmitoylation and signaling. *Proc Natl Acad Sci U S A* 115(33)**,** E7768-e7775. doi: 10.1073/pnas.1806239115.

Hanson, M.C., Crespo, M.P., Abraham, W., Moynihan, K.D., Szeto, G.L., Chen, S.H., et al. (2015). Nanoparticulate STING agonists are potent lymph node-targeted vaccine adjuvants. *J Clin Invest* 125(6)**,** 2532-2546. doi: 10.1172/jci79915.

Hong, Z., Mei, J., Li, C., Bai, G., Maimaiti, M., Hu, H., et al. (2021). STING inhibitors target the cyclic dinucleotide binding pocket. *Proc Natl Acad Sci U S A* 118(24). doi: 10.1073/pnas.2105465118.

Huang, K.C., Chanda, D., McGrath, S., Dixit, V., Zhang, C., Wu, J., et al. (2022). Pharmacologic Activation of STING in the Bladder Induces Potent Antitumor Immunity in Non-Muscle Invasive Murine Bladder Cancer. *Mol Cancer Ther* 21(6)**,** 914-924. doi: 10.1158/1535-7163.Mct-21-0780.

Humphries, F., Shmuel-Galia, L., Jiang, Z., Wilson, R., Landis, P., Ng, S.L., et al. (2021). A diamidobenzimidazole STING agonist protects against SARS-CoV-2 infection. *Sci Immunol* 6(59). doi: 10.1126/sciimmunol.abi9002.

Jing, W., McAllister, D., Vonderhaar, E.P., Palen, K., Riese, M.J., Gershan, J., et al. (2019). STING agonist inflames the pancreatic cancer immune microenvironment and reduces tumor burden in mouse models. *J Immunother Cancer* 7(1)**,** 115. doi: 10.1186/s40425-019-0573-5.

Kim, D.S., Endo, A., Fang, F.G., Huang, K.C., Bao, X., Choi, H.W., et al. (2021). E7766, a Macrocycle-Bridged Stimulator of Interferon Genes (STING) Agonist with Potent Pan-Genotypic Activity. *ChemMedChem* 16(11)**,** 1740-1743. doi: 10.1002/cmdc.202100068.

Lai, J., Luo, X., Tian, S., Zhang, X., Huang, S., Wang, H., et al. (2020). Compound C Reducing Interferon Expression by Inhibiting cGAMP Accumulation. *Front Pharmacol* 11**,** 88. doi: 10.3389/fphar.2020.00088.

Lara, P.N., Jr., Douillard, J.Y., Nakagawa, K., von Pawel, J., McKeage, M.J., Albert, I., et al. (2011). Randomized phase III placebo-controlled trial of carboplatin and paclitaxel with or without the vascular disrupting agent vadimezan (ASA404) in advanced non-small-cell lung cancer. *J Clin Oncol* 29(22)**,** 2965-2971. doi: 10.1200/jco.2011.35.0660.

Li, L., Yin, Q., Kuss, P., Maliga, Z., Millán, J.L., Wu, H., et al. (2014). Hydrolysis of 2'3'-cGAMP by ENPP1 and design of nonhydrolyzable analogs. *Nat Chem Biol* 10(12)**,** 1043-1048. doi: 10.1038/nchembio.1661.

Li, S., Hong, Z., Wang, Z., Li, F., Mei, J., Huang, L., et al. (2018). The Cyclopeptide Astin C Specifically Inhibits the Innate Immune CDN Sensor STING. *Cell Rep* 25(12)**,** 3405-3421.e3407. doi: 10.1016/j.celrep.2018.11.097.

Li, S., Luo, M., Wang, Z., Feng, Q., Wilhelm, J., Wang, X., et al. (2021). Prolonged activation of innate immune pathways by a polyvalent STING agonist. *Nat Biomed Eng* 5(5)**,** 455-466. doi: 10.1038/s41551-020-00675-9.

Liu, Y., Xiao, N., Du, H., Kou, M., Lin, L., Huang, M., et al. (2020). Celastrol ameliorates autoimmune disorders in Trex1-deficient mice. *Biochem Pharmacol* 178**,** 114090. doi: 10.1016/j.bcp.2020.114090.

Liu, Z., Zhou, J., Xu, W., Deng, W., Wang, Y., Wang, M., et al. (2022). A novel STING agonist-adjuvanted pan-sarbecovirus vaccine elicits potent and durable neutralizing antibody and T cell responses in mice, rabbits and NHPs. *Cell Res* 32(3)**,** 269-287. doi: 10.1038/s41422-022-00612-2.

Luo, X., Li, H., Ma, L., Zhou, J., Guo, X., Woo, S.L., et al. (2018). Expression of STING Is Increased in Liver Tissues From Patients With NAFLD and Promotes Macrophage-Mediated Hepatic Inflammation and Fibrosis in Mice. *Gastroenterology* 155(6)**,** 1971-1984.e1974. doi: 10.1053/j.gastro.2018.09.010.

Meric-Bernstam, F., Sweis, R.F., Hodi, F.S., Messersmith, W.A., Andtbacka, R.H.I., Ingham, M., et al. (2022). Phase I Dose-Escalation Trial of MIW815 (ADU-S100), an Intratumoral STING Agonist, in Patients with Advanced/Metastatic Solid Tumors or Lymphomas. *Clin Cancer Res* 28(4)**,** 677-688. doi: 10.1158/1078-0432.Ccr-21-1963.

Ming, S.L., Zeng, L., Guo, Y.K., Zhang, S., Li, G.L., Ma, Y.X., et al. (2020). The Human-Specific STING Agonist G10 Activates Type I Interferon and the NLRP3 Inflammasome in Porcine Cells. *Front Immunol* 11**,** 575818. doi: 10.3389/fimmu.2020.575818.

Pan, B.S., Perera, S.A., Piesvaux, J.A., Presland, J.P., Schroeder, G.K., Cumming, J.N., et al. (2020). An orally available non-nucleotide STING agonist with antitumor activity. *Science* 369(6506). doi: 10.1126/science.aba6098.

Peng, Y., Zhuang, J., Ying, G., Zeng, H., Zhou, H., Cao, Y., et al. (2020). Stimulator of IFN genes mediates neuroinflammatory injury by suppressing AMPK signal in experimental subarachnoid hemorrhage. *J Neuroinflammation* 17(1)**,** 165. doi: 10.1186/s12974-020-01830-4.

Prabakaran, T., Troldborg, A., Kumpunya, S., Alee, I., Marinković, E., Windross, S.J., et al. (2021). A STING antagonist modulating the interaction with STIM1 blocks ER-to-Golgi trafficking and inhibits lupus pathology. *EBioMedicine* 66**,** 103314. doi: 10.1016/j.ebiom.2021.103314.

Ramanjulu, J.M., Pesiridis, G.S., Yang, J., Concha, N., Singhaus, R., Zhang, S.Y., et al. (2018). Design of amidobenzimidazole STING receptor agonists with systemic activity. *Nature* 564(7736)**,** 439-443. doi: 10.1038/s41586-018-0705-y.

Rech, L., Abdellatif, M., Pöttler, M., Stangl, V., Mabotuwana, N., Hardy, S., et al. (2022). Small molecule STING inhibition improves myocardial infarction remodeling. *Life Sci* 291**,** 120263. doi: 10.1016/j.lfs.2021.120263.

Sali, T.M., Pryke, K.M., Abraham, J., Liu, A., Archer, I., Broeckel, R., et al. (2015). Characterization of a Novel Human-Specific STING Agonist that Elicits Antiviral Activity Against Emerging Alphaviruses. *PLoS Pathog* 11(12)**,** e1005324. doi: 10.1371/journal.ppat.1005324.

Shen, R., Yang, K., Cheng, X., Guo, C., Xing, X., Sun, H., et al. (2022). Accumulation of polystyrene microplastics induces liver fibrosis by activating cGAS/STING pathway. *Environ Pollut* 300**,** 118986. doi: 10.1016/j.envpol.2022.118986.

Song, C., Liu, D., Liu, S., Li, D., Horecny, I., Zhang, X., et al. (2022). SHR1032, a novel STING agonist, stimulates anti-tumor immunity and directly induces AML apoptosis. *Sci Rep* 12(1)**,** 8579. doi: 10.1038/s41598-022-12449-1.

Wang, J., Li, P., Yu, Y., Fu, Y., Jiang, H., Lu, M., et al. (2020). Pulmonary surfactant-biomimetic nanoparticles potentiate heterosubtypic influenza immunity. *Science* 367(6480). doi: 10.1126/science.aau0810.

Xu, Q., Xiong, H., Zhu, W., Liu, Y., and Du, Y. (2020). Small molecule inhibition of cyclic GMP-AMP synthase ameliorates sepsis-induced cardiac dysfunction in mice. *Life Sci* 260**,** 118315. doi: 10.1016/j.lfs.2020.118315.

Zhang, X., Liu, B., Tang, L., Su, Q., Hwang, N., Sehgal, M., et al. (2019). Discovery and Mechanistic Study of a Novel Human-Stimulator-of-Interferon-Genes Agonist. *ACS Infect Dis* 5(7)**,** 1139-1149. doi: 10.1021/acsinfecdis.9b00010.

Zhang, Y., Sun, Z., Pei, J., Luo, Q., Zeng, X., Li, Q., et al. (2018). Identification of α-Mangostin as an Agonist of Human STING. *ChemMedChem* 13(19)**,** 2057-2064. doi: 10.1002/cmdc.201800481.

Zhao, Q., Wei, Y., Pandol, S.J., Li, L., and Habtezion, A. (2018). STING Signaling Promotes Inflammation in Experimental Acute Pancreatitis. *Gastroenterology* 154(6)**,** 1822-1835.e1822. doi: 10.1053/j.gastro.2018.01.065.
